# Loss of SMARCB1 evokes targetable epigenetic vulnerabilities in epithelioid sarcoma

**DOI:** 10.1002/cac2.12665

**Published:** 2025-01-20

**Authors:** Jia Xiang Jin, Fabia Fuchslocher, Martha Carreno‐Gonzalez, Felina Zahnow, A. Katharina Ceranski, Rainer Will, Dominic Helm, Felix Bestvater, Ana Banito, Roland Imle, Shunya Ohmura, Florencia Cidre‐Aranaz, Thomas G. P. Grünewald

**Affiliations:** ^1^ Hopp‐Children's Cancer Center (KiTZ) Heidelberg Baden‐Württemberg Germany; ^2^ Division of Translational Pediatric Sarcoma Research (B410) German Cancer Research Center (DKFZ) German Cancer Consortium (DKTK) Heidelberg Baden‐Württemberg Germany; ^3^ Medical Faculty Ruprecht‐Karls‐University Heidelberg Baden‐Württemberg Germany; ^4^ National Center for Tumor Diseases (NCT) NCT Heidelberg a partnership between DKFZ and Heidelberg University Hospital Heidelberg Baden‐Württemberg Germany; ^5^ Core Facility Cellular Tools (W111) German Cancer Research Center (DKFZ) German Cancer Consortium (DKTK) Heidelberg Baden‐Württemberg Germany; ^6^ Core Facility Proteomics (W120) German Cancer Research Center (DKFZ) German Cancer Consortium (DKTK) Heidelberg Baden‐Württemberg Germany; ^7^ Light Microscopy Core Facility (W210) German Cancer Research Center (DKFZ) German Cancer Consortium (DKTK) Heidelberg Baden‐Württemberg Germany; ^8^ Soft‐Tissue Sarcoma Junior Research Group German Cancer Research Center (DKFZ) German Cancer Consortium (DKTK) Heidelberg Baden‐Württemberg Germany; ^9^ Faculty of Biosciences Heidelberg University Heidelberg Baden‐Württemberg Germany; ^10^ Division of Pediatric Surgery Department of General Visceral and Transplantation Surgery University Hospital Heidelberg Heidelberg Baden‐Württemberg Germany; ^11^ Institute of Pathology Heidelberg University Hospital Heidelberg Baden‐Württemberg Germany

**List of abbreviations**
AP‐1: Activator Protein‐1
ARID1B: AT‐Rich Interaction Domain 1B
ATAC‐Seq: Assay for Transposase‐Accessible Chromatin using Sequencing
ATF2: Activating Transcription Factor 2
BRG1: Brahma‐Related Gene 1
cBAF: Canonical BRG1/BRM‐associated factor
ChIP‐Seq: Chromatin immunoprecipitation followed by DNA‐sequencing
Co‐IP: Co‐immunoprecipitation
DCAF5: DDB1 And CUL4 Associated Factor 5
DMSO: Dimethyl‐sulfoxide
DOX: Doxycycline
EpS: Epithelioid sarcoma
EwS: Ewing sgarcoma
ESR1/ESR2: Estrogen Receptor 1/2
EZH2: Enhancer of zeste homology 2
FOSL1: Fos Like 1
GREAT: Genomic Regions Enrichment of Annotations Tool
GSEA: Gene Set Enrichment Analysis
H&E: Hematoxylin/eosin
HPF: High Power Field
JUND: Jun D Proto‐Oncogene
KLF8: Krüppel‐Like Factor 8
MYC: Proto‐Oncogene C‐Myc
ncBAF: Non‐canonical BRG1/BRM‐associated factor
NFKB1: Nuclear Factor Kappa B Subunit 1
NSG: Nod/Scid/gamma
nTPM: normalized transcripts per million
PBAF: Polybromo‐associated BRG1/BRM‐associated factor
PI: Propidium Iodide
POU5F1: POU Class 5 Homeobox 1
RAD21: RAD21 Cohesin Complex Component
s.c.: subcutaneous
sCRE: SWI/SNF‐specific cis‐regulatory elements
SEA: Simple motif Enrichment Analyses
SMARCB1: SWI/SNF‐related matrix‐associated actin‐dependent regulator of chromatin subfamily B member 1
STC1: Stanniocalcin‐1
SWI/SNF: SWItch/Sucrose Non‐Fermentable
rSWI/SNF: residual SWItch/Sucrose Non‐Fermentable
TGFβ: Transforming growth factor beta
TGFBI: Transforming growth factor beta induced
TF: Transcription factor
TSS: Transcription start site
qPCR: Quantitative Polymerase Chain Reaction
VEZF1: Vascular Endothelial Zinc Finger 1
WGCNA: Weighted Gene Correlation Network Analysis

1

Epithelioid sarcoma (EpS) is a high‐grade malignancy of unknown histogenesis first described in 1970 [1], characterized by high rates of relapse and metastasis, with 5‐year survival rates of 60%‐75% [2]. The only Food and Drug Administration (FDA)‐approved targeted therapy, the enhancer of zeste homology 2 (EZH2) inhibitor tazemetostat, achieved transient responses in only 15% of patients [2]. To establish a solid mechanistic basis, we investigated the role of SWI/SNF related BAF chromatin remodeling complex subunit B1 (*SMARCB1*) via multi‐omics analyses. We engineered isogenic cell line models single‐cell‐cloned to minimize genetic variability, featuring doxycycline‐(DOX)‐inducible *SMARCB1* expression systems alongside respective empty vector controls. The cell lines (FU‐EPS‐1; HS‐ES‐1, ‐2M, ‐2R; NEPS; VA‐ES‐BJ) exhibited homozygous *SMARCB1* deletion and represented proximal and distal subtypes, with prominent *SMARCB1* re‐expression upon DOX‐treatment (Figure 1A). DOX concentrations were adjusted to achieve SYBR/TaqMan‐qPCR‐controlled *SMARCB1* levels comparable to *SMARCB1*‐proficient Ewing sarcoma (EwS) cell lines, minimizing experimental artefacts associated with supraphysiological expression (Supplementary Figure S1A‐B). Western blots demonstrated that SMARCB1 underwent nuclear translocation and re‐incorporation into the SWI/SNF complex (Figure 1B). Transcriptome profiling using Affymetrix Clariom D microarrays (GEO: GSE276634) and Weighted Gene Correlation Network Analysis (WGCNA) based on Gene Set Enrichment Analysis (GSEA) revealed downregulated signatures related to DNA‐repair and epigenetic regulation, alongside upregulated developmental pathways upon *SMARCB1* re‐expression (Figure 1C). These findings were accompanied by dose‐dependent reductions in clonogenicity (Figure 1D, Supplementary Figure S1C), while propidium‐iodide‐(PI)‐based flow‐cytometric cell‐cycle‐analysis showed delayed G1‐to‐S‐phase transition (Supplementary Figure S1D). Orthotopic subcutaneous (s.c.) xenotransplantation experiments using VA‐ES‐BJ in immunocompromised *Nod/Scid/gamma* (NSG) mice recapitulated the typical EpS morphology (Supplementary Figure S1E). After tumors became palpable, *SMARCB1* re‐expression via DOX supplementation in drinking water resulted in significantly reduced tumor growth (Figure 1E).

**FIGURE 1:**
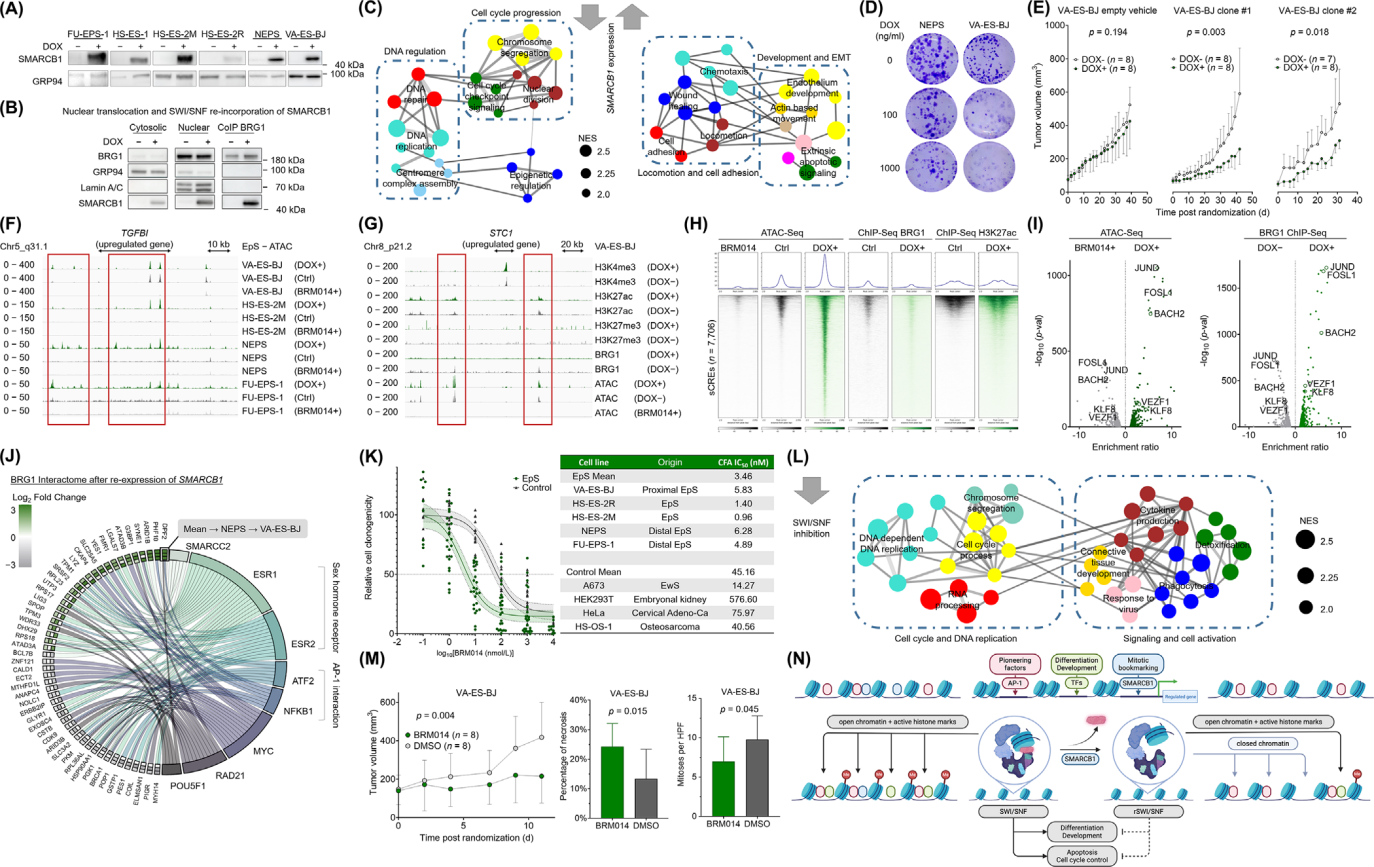
Multi‐omics functional analysis of *SMARCB1* re‐expression in isogenic EpS models reveals rSWI/SNF as a targetable epigenetic vulnerability. (A) Western blot of clonal EpS cell lines re‐expressing SMARCB1, demonstrating high re‐expressional efficiency and low vector leakiness. (B) Western blot of cytosolic, nuclear, and BRG1‐co‐immunoprecipitated cell lysates (VA‐ES‐BJ) probed for BRG1, GRP94 (cytosolic marker), Lamin A/C (nuclear marker), and SMARCB1. SMARCB1 relocates to the nuclear compartment and re‐integrates into the SWI/SNF complex. (C) GSEA‐based network analysis of upregulated and downregulated gene sets following SMARCB1 re‐expression in VA‐ES‐BJ and NEPS, showing activation of differentiation‐associated pathways in lieu of proliferation‐associated pathways. (D) Dosage‐dependent loss of clonogenicity in VA‐ES‐BJ and NEPS upon DOX treatment, showing a gene‐dose‐dependent action for *SMARCB1*. (E) In vivo tumor growth curves of VA‐ES‐BJ subcutaneous xenografts (empty vehicle or isogenic models with *SMARCB1* re‐expression system) treated with either empty vehicle or DOX, demonstrating SMARCB1‐mediated tumor growth inhibition. (F) Example ATAC‐Seq tracks (representative merged replicates) of EpS cell lines at the *TGFBI* locus showing chromatin opening in the DOX+ condition and closing under BRM014 treatment. The left box highlights regulated enhancer regions upstream of *TGFBI*, while the right box shows chromatin accessibility at the *TGFBI* gene body (*n* = 2 biological replicates per condition). (G) Example ChIP‐Seq and ATAC‐Seq tracks (representative merged replicates) at the *STC1* gene locus in VA‐ES‐BJ, showing differential histone marks and open chromatin distribution correlating with upregulated gene expression in transcriptomic data (*n* = 2 biological replicates per condition). (H) Heatmaps of BRG1, H3K27ac (active enhancer mark) occupancy, and chromatin accessibility (representative replicates) across loci with differential ATAC‐Seq enrichment (*n* = 2 biological replicates per condition). (I) Volcano plots of Simple Enrichment Analysis (SEA) showing enriched motifs in open chromatin regions lost upon BRM014 treatment and gained upon *SMARCB1* re‐expression (pooled EpS cell lines). BRG1 ChIP sites with and without *SMARCB1* re‐expression are shown for VA‐ES‐BJ. (J) Chord diagram showing proteins co‐immunoprecipitating with the core SWI/SNF‐ATPase BRG1 that are significantly regulated upon *SMARCB1* re‐expression in NEPS and VA‐ES‐BJ (*n* = 4 biological replicates). Log_2_ fold changes (left) are presented as concentric annuli (outermost to innermost: Mean, NEPS, VA‐ES‐BJ). Enrichr‐based transcription factor protein‐protein interactions are depicted on the right. Left–right connections indicate gene/protein membership in the interactome of a transcription factor. (K) Pooled BRM014 drug response curves with 95% confidence intervals (CI) and individual IC_50_ values for SWI/SNF‐deficient EpS cell lines (FU‐EPS‐1, HS‐ES‐2M, HS‐ES‐2R, NEPS, VAESBJ) versus SWI/SNF wild‐type control lines (HEK293T, HeLa, A‐673, HS‐OS‐1), demonstrating a significant therapeutic window for (r)SWI/SNF targeting in EpS. (L) GSEA‐based network analysis of downregulated gene sets upon residual SWI/SNF inhibition by BRM014 treatment in HS‐ES‐2M, HS‐ES‐2R and NEPS. (M) Growth curves of VA‐ES‐BJ xenografts treated with BRM014 or vehicle control (DMSO). Bar plots show percentage of necrosis and mitoses per high‐power field (HPF) observed in histological analysis, demonstrating in vivo efficacy of (r)SWI/SNF inhibition. (N) Schematic representation of residual SWI/SNF function in EpS. Pioneering factors, mitotic bookmarkers, and specific transcription factors pre‐assemble at sCREs, recruiting physiological SMARCB1‐proficient SWI/SNF to regulate differentiation and cell cycle control. Loss of SMARCB1 significantly disrupts this balance, leading to closed chromatin and decreased active histone marks at specific sCREs, driving tumorigenesis. Abbreviations: EpS, Epithelioid Sarcoma; SMARCB1, SWI/SNF‐related matrix‐associated actin‐dependent regulator of chromatin subfamily B member 1; BRG1, Brahma‐Related Gene 1; Co‐IP, Co‐immunoprecipitation; GSEA, Gene Set Enrichment Analysis; DOX, Doxycycline; s.c., subcutaneous; ATAC‐Seq, Assay for Transposase‐Accessible Chromatin using Sequencing; TGFBI, Transforming growth factor beta induced; ChIP‐Seq, Chromatin immunoprecipitation followed by DNA‐sequencing; STC1, Stanniocalcin‐1; SEA, Simple motif Enrichment Analyses; HPF, High Power Field; DMSO, Dimethyl sulfoxide; (r)SWI/SNF, (residual) SWItch/Sucrose Non‐Fermentable; TF, Transcription Factor; sCRE, SWI/SNF‐specific cis‐regulatory element.

Since these findings underscored significant *SMARCB1*‐associated epigenetic regulation (Figure 1C) [3], we next investigated SWI/SNF chromatin‐remodeling functionality via Assay for Transposase‐Accessible Chromatin using Sequencing (ATAC‐Seq; GEO: GSE281434) in FU‐EPS‐1, HS‐ES‐2M, NEPS and VA‐ES‐BJ to compare the effects of *SMARCB1*‐deficient and physiological SWI/SNF assemblies. *SMARCB1* re‐expression increased chromatin accessibility at putative enhancer sites (box 1) and gene bodies (box 2) (Figure 1F). Conversely, SWI/SNF‐inhibition using BRM014 (Compound‐14), a small‐molecule allosteric dual SWI/SNF‐ATPase inhibitor, resulted in decreased chromatin accessibility at these sites (Supplementary Figure S2A). While most cell lines showed chromatin opening at both, in VA‐ES‐BJ, chromatin opening occurred preferentially at upstream regulatory regions near the *TGFBI* locus, with only minor opening at the gene body, highlighting subtle subtype‐dependent biological differences. Next, we performed Chromatin immunoprecipitation followed by DNA‐sequencing (ChIP‐Seq; GEO: GSE281436) in VA‐ES‐BJ, probing for SWI/SNF subunits (BRG1 and SMARCB1) and histone‐marks indicative of active enhancers (H3K27ac), active promoters (H3K4me3), and polycomb repression (H3K27me3) to elucidate functional chromatin status. *SMARCB1* re‐expression led to increases in H3K4me3/H3K27ac‐occupancy, demonstrating tight SWI/SNF‐mediated regulation (Figure 1G). These differentially accessible regions likely represent SWI/SNF‐specific *cis*‐regulatory‐elements (sCRE) driving functional changes through the reactivation of enhancer histone‐marks (Figure 1H). BRG1‐occupancy, initially restricted to a subset of sCRE, was redistributed more broadly after *SMARCB1* re‐expression (Figure 1H). Simple motif Enrichment Analyses (SEA) showed that lost and regained sCRE motifs belonged to similar transcription factor (TF) families (Figure 1I). Enrichment‐ratios for Activator Protein‐1 (AP‐1) TFs (e.g., JUND, FOSL1) increased significantly following *SMARCB1* re‐expression, while development‐associated TFs (e.g., VEZF1, KLF8) showed less pronounced but notable enrichment increases. This mirrored SEA of differentially bound BRG1 sites (Figure 1I), aligning with AP‐1 TFs acting as pioneering factors facilitating epigenetic restructuring via SWI/SNF cooperation [4, 5]. Differential SEA of sCREs indicated that motifs associated with cell cycle progression and apoptosis were lost upon BRM014 treatment, while developmental and homeobox motifs were gained following *SMARCB1* re‐expression (Supplementary Figure S2B). Residual SWI/SNF‐sites (BRG1‐DOX−) were linked to proliferation, whereas SMARCB1‐associated motifs (SMARCB1‐DOX+) were associated with development (Supplementary Figure S2C). This epigenetic shift may represent the re‐activation of lineage‐dependent developmental pathways via SMARCB1‐mediated bookmarking functions [6]. Further, we found increased percentages of bivalent and polycomb‐repressed (H3K27me3) promotors at gained distal (up to 1 Mb) but not proximal (≤ 2 kb) BRG1‐associated genes, suggesting preferentially distal epigenetic restructuring (Supplementary Figure S3A) [7].

Global chromatin accessibility showed the closest correlation within the same subtype, suggesting a proximal origin for the HS‐ES‐2 models, which lack histological metadata. BRM014‐treated sCREs clustered together across models (Supplementary Figure S3B), indicating that these sCREs may represent highly conserved sites intricately involved in tumor maintenance. Genomic Regions Enrichment of Annotations Tool (GREAT) analysis of differentially bound histone‐marks and BRG1‐loci showed associations with the regulation of apoptosis and developmental pathways (Supplementary Figure S4A). GREAT analyses indicated that BRM014‐specific sCREs were apoptosis‐associated, while *SMARCB1*‐specific sCREs exhibited subtype‐dependent development‐associated signatures (Supplementary Figure S4B‐D). Preserved TGFβ‐signaling across sCRE groups potentially highlights it as a central pathway in EpS. Overall, the increased chromatin accessibility and change in histone‐marking at sCREs are likely functionally related to SWI/SNF‐mediated TF‐recruitment, followed by subsequent epigenetic modulation. This presumably activates signaling cascades that converge in the upregulation of differentiation‐associated pathways. These pathways, in turn, interact with and downregulate mutually exclusive pathways not directly occupied by SWI/SNF, as evidenced by GREAT analysis of ATAC‐sites showing both negative and positive pathway regulation (Supplementary Figure S4).

Next, we performed mass‐spectrometry‐based quantification of BRG1‐co‐immunoprecipitated nuclear proteins (PRIDE: PXD053945) in NEPS and VA‐ES‐BJ to elucidate *SMARCB1*‐associated changes in the SWI/SNF proteome and interactome. Enrichr‐based TF protein‐protein‐interaction enrichment analysis demonstrated increased interaction with other SWI/SNF subunits (e.g., ARID1B) upon *SMARCB1* re‐expression, suggesting SWI/SNF assembly dependence on *SMARCB1* status (Figure 1J). The influence of the AP‐1 and sex hormonal TFs was evident in the regulated interactomes of ATF2, NFKB1 and ESR1/ESR2. Other enriched TFs were associated with proliferation, chromatin organization, and cell fate determination (e.g., MYC, RAD21, POU5F1) (Figure 1J). GSEA revealed that *SMARCB1* re‐expression downregulated chromosome organization and telomere maintenance, while upregulating energy metabolism and development signatures (Supplementary Figure S5A). Global proteomics highlighted a downregulation of DNA/RNA regulation and translation, whereas development‐ and cytoskeleton‐associated proteins were upregulated (Supplementary Figure S5B). Overall, these significant changes to the SWI/SNF assembly and interactome may enhance chromatin modulation, explaining the increased chromatin accessibility at BRG1‐associated sites, despite a general decrease in BRG1 binding (Figure 1G, H, and J).

Given these results in support of the residual SWI/SNF (rSWI/SNF) complex as a druggable target, we performed clonogenic growth assays and drug‐response assays with BRM014. These assays demonstrated dose‐dependent reductions in clonogenicity and cell proliferation (Figure 1K, Supplementary Figure S6A‐I) [8]. BRM014, based on X‐ray‐crystallographic data, is an allosteric dual SWI/SNF‐ATPase inhibitor [9]. Although these data do not suggestBRM014‐associated alterations to SWI/SNF assembly, future studies are necessary to investigate this possibility (Figure 1J). The short‐term effects of BRM014 on cell proliferation were modest (Supplementary Figure S6J), consistent with delayed epigenetic remodeling and the absence of any microscopically detectable changes in cell morphology/confluency. WGCNA upon BRM014‐treatment revealed downregulated signatures involved in DNA‐replication, cell cycle progression and cytokine production (Figure 1L). One significantly upregulated gene set (negative regulation of muscle cell differentiation, NES = 2) was identified. To control for assembly‐specific SWI/SNF interactions, we performed drug‐assays with dBRD9 [10], which selectively degrades BRD9 – a core subunit exclusive to ncBAF, lacking SMARCB1. No effects were observed on cell proliferation, indicating that the ATPase function of SMARCB1‐containing cBAF and PBAF were most likely essential for tumor survival in EpS (Supplementary Figure S6K). While our findings do not exclude the potential involvement of the ncBAF complex in tumor maintenance in EpS, they suggest that the tumorigenic functionalities of the rSWI/SNF complex can be effectively abrogated by targeting the therapeutically more accessible SWI/SNF‐ATPases.

To evaluate the in vivo potential of SWI/SNF‐ATPase inhibition as a novel targeted approach, we treated subcutaneous xenograft NSG mouse models with intraperitoneal injections of BRM014 [11]. This treatment significantly suppressed tumor growth, decreased mitoses per HPF, and increased necrosis (Figure 1M, Supplementary Figure S6L). This aligned with mechanistic insights demonstrated in our and published data [4, 6, 7] (Figure 1N). GSEA demonstrated that SWI/SNF‐inhibition and *SMARCB1* re‐expression triggered a comparable loss of immature cell signatures (Supplementary Figure S7A‐B). EnrichR‐based MSigDB‐hallmark pathway analysis of shared regulated genes after *SMARCB1* re‐expression and BRM014‐treatment revealed shared downregulation of cell cycle progression and MYC‐associated proliferation, alongside upregulation of EMT processes, likely differentiation‐related in EpS (Supplementary Figure S7C‐D).

These similarities prompted us to explore synergistic effects between *SMARCB1* re‐expression and rSWI/SNF‐inhibition. Clonogenicity‐based combination drug assays in VA‐ES‐BJ provided evidence of significant synergy (Supplementary Figure S8A‐C). Bliss scores increased with dosages of DOX and BRM014, reaching a saturated plateau at 1 µg/ml DOX and 1 nmol/L BRM014. This drastically reduced the doses of both compounds required to elicit significant loss of clonogenicity. While both epithelioid and mesenchymal‐like cell morphologies—characteristic for EpS—were present at baseline (shown exemplarily for VA‐ES‐BJ in Supplementary Figure S8D), *SMARCB1* re‐expression resulted in a morphological shift towards more mesenchymal‐like phenotypes. These changes potentially reflected differentiation‐associated biological processes discovered in our multi‐omics analyses (Figures 1C and L, Supplementary Figure S4, Supplementary Figure S7). Although *SMARCB1* re‐expression is not currently a feasible therapeutic approach, alternative strategies—such as the inhibition of DCAF5‐mediated SWI/SNF‐degradation—may exploit similar mechanisms and represent promising combination partners for SWI/SNF ATPase inhibition [5].

Contrary to expectations, our findings underscore SWI/SNF‐targeting as a viable therapeutic approach for EpS, despite its hallmark SWI/SNF‐deficiency. This discovery may motivate further investigations into whether similar targeted strategies could be effective in other SWI/SNF‐deficient entities.

## AUTHOR CONTRIBUTIONS

Jia Xiang Jin established isogenic cell line models, designed and performed functional in vitro andin vivo experiments including bioinformatic and histological analyses. Florencia Cidre‐Aranaz performed in vivo experiments. Fabia Fuchslocher carried out in vitro experiments and established isogenic cell line models. Felina Zahnow assisted in in vitro experiments. Martha Carreno‐Gonzalez, Shunya Ohmura, Ana Banito and Roland Imle assisted in in vivo experiments and A. Katharina Ceranski in histological analysis. Rainer Will assisted in the generation of cell line models. Dominic Helm provided expertise in mass‐spectrometric proteome measurement. Felix Bestvater assisted in the generation of histological stains. Thomas G. P. Grünewald designed and supervised the study, provided biological and technical guidance as well as laboratory infrastructure. All authors read and approved the final manuscript.

## CONFLICT OF INTEREST STATEMENT

The authors declare no conflict of interest.

## FUNDING INFORMATION

This project was mainly supported by a grant from the SMARCB1 association. The laboratory of Thomas G. P. Grünewald is further supported by grants from the Dr. Rolf M. Schwiete foundation (2021‐007, 2022‐031), the Matthias‐Lackas foundation, the Dr. Leopold und Carmen Ellinger foundation, the Deutsche Forschungsgemeinschaft (DFG 458891500), the Cancer Grand Challenges project PROTECT, the German Cancer Aid (DKH‐7011411, DKH‐70114278, DKH‐70115315, DKH‐70115914), the Ministry of Education and Research (BMBF; SMART‐CARE and HEROES‐AYA), the KiKa foundation (#486), the Fight Kids Cancer foundation (FKC‐NEWtargets), the KiTZ‐Foundation in memory of Kirstin Diehl, the KiTZ‐PMC twinning program, the German Cancer Consortium (DKTK, PRedictAHR), and the Barbara and Wilfried Mohr foundation. The laboratory of Thomas G. P. Grünewald is co‐funded by the European Union (ERC, CANCER‐HARAKIRI, 101122595). Views and opinions expressed are solely those of the authors and do not necessarily reflect those of the European Union or the European Research Council. Neither the European Union nor the granting authority can be held responsible for them. Jia Xiang Jin and Fabia Fuchslocher were supported by scholarships from the Rudolf and Brigitte Zenner foundation and the German Academic Scholarship Foundation.

### ETHICS APPROVAL AND CONSENT TO PARTICIPATE

Animal experiments were conducted with approval from the government of North Baden (NTP‐ID: 00029631‐1‐6) and in compliance with the 3R principle of animal experiments (replacement, reduction, and refinement). The study adhered to the ARRIVE guidelines, the recommendations of the European Community (86/609/EEC), and the UKCCCR (guidelines for the welfare and use of animals in cancer research).

## Supporting information



Supporting Information

## Data Availability

Original Affymetrix transcriptome profiling data have been deposited in the Gene Expression Omnibus (GEO) under the accession code GSE276634. Proteomics data have been deposited in the Proteomics Identifications Database (PRIDE) under accession code PXD053945. ATAC‐Seq and ChIP‐Seq data have been deposited in GEO under the accession series codes GSE281434 and GSE281436.
