# Supplementary material for: Loss of SMARCB1 evokes targetable epigenetic vulnerabilities in epithelioid sarcoma
Source: Cancer Commun (Lond). 2025 Jan 20;45(5):494–9. doi: 10.1002/cac2.12665 (PMC12067397; doi:10.1002/cac2.12665)
Supplement: Supplementary file 1 — Supporting Information [file CAC2-45-494-s001.docx]

**Supplementary Materials**

**Loss of *SMARCB1* evokes targetable epigenetic vulnerabilities in epithelioid sarcoma**

Jia Xiang Jin^1,2,3,4^, Fabia Fuchslocher^1,2,3,4^, Martha Carreno-Gonzalez^1,2,3,4^, Felina Zahnow^1,2,4^, A. Katharina Ceranski^1,2,3,4^, Rainer Will^5^, Dominic Helm^6^, Felix Bestvater^7^, Ana Banito^1,4,8^, Roland Imle^1,4,8,9,10^, Shunya Ohmura^1,2,4^, Florencia Cidre-Aranaz^1,2,4^, Thomas G. P. Grünewald^1,2,4,11,*^

^1^ Hopp-Children’s Cancer Center (KiTZ), Heidelberg, BW, Germany.

^2^ Division of Translational Pediatric Sarcoma Research (B410), German Cancer Research Center (DKFZ), German Cancer Consortium (DKTK), Heidelberg, BW, Germany.

^3^ Medical Faculty, Ruprecht-Karls-University, Heidelberg, BW, Germany.

^4^ National Center for Tumor Diseases (NCT), NCT Heidelberg, a partnership between DKFZ and Heidelberg University Hospital, Heidelberg, BW, Germany.

^5^ Core Facility Cellular Tools (W111), German Cancer Research Center (DKFZ), German Cancer Consortium (DKTK), Heidelberg, BW, Germany.

^6^ Core Facility Proteomics (W120), German Cancer Research Center (DKFZ), German Cancer Consortium (DKTK), Heidelberg, BW, Germany.

^7^ Light Microscopy Core Facility (W210), German Cancer Research Center (DKFZ), German Cancer Consortium (DKTK), Heidelberg, BW, Germany.

^8^ Soft-Tissue Sarcoma Junior Research Group, German Cancer Research Center (DKFZ), German Cancer Consortium (DKTK), Heidelberg, BW, Germany.

^9^ Faculty of Biosciences, Heidelberg University, Heidelberg, BW, Germany.

^10^ Division of Pediatric Surgery, Department of General, Visceral and Transplantation Surgery, University Hospital Heidelberg, Heidelberg, BW, Germany.

^11^ Institute of Pathology, Heidelberg University Hospital, Heidelberg, BW, Germany.

^*^**Corresponding author:**

Thomas G. P. Grünewald; Division of Translational Pediatric Sarcoma Research

German Cancer Research Center (DKFZ); Im Neuenheimer Feld 280, 69120 Heidelberg, Germany; Tel.: +49-69221-42-3718; Email: t.gruenewald@dkfz-heidelberg.de.

**Supplementary Materials and Methods**

**Provenience of cell lines and culture conditions**

Human Epithelioid Sarcoma (EpS) cell lines were obtained from the following repositories/providers: HE-ES-1, HS-ES-2M, and HS-ES-2R from RIKEN cell bank (Wako, Saitama, Japan); VA-ES-BJ from the German Collection of Microorganisms and Cell cultures (DSMZ, Braunschweig, Germany). NEPS from Niigata University (Japan); FU-EPS-1 (ST257) from Fukuoka University (Japan); and Epi-544 from MD Anderson Cancer Center, University of Texas (Houston, TX, USA). All EpS cell lines exhibited virtually no detectable *SMARCB1* expression (Ct_q_ values > 35 in TaqMan qPCR). Human Ewing sarcoma (EwS) cell lines were obtained from the following repositories/providers: A-673 from American Type Culture Collection (ATCC, Manassas, VA, USA); MHH-ES1 and SK-N-MC from DSMZ; TC-106 and TC-71 from the Children’s Oncology Group (COG). The human osteosarcoma cell line HS-OS-1 was obtained from RIKEN cell bank (Wako, Saitama, Japan). Human HEK293T and HeLa cells were obtained from DSMZ. All cell lines were cultured in RPMI 1640 with stable glutamine (Gibco, Thermo Fisher, Waltham, MA, USA), supplemented with 10% fetal bovine serum (FBS), 100 U/ml penicillin, and 100 μg/ml streptomycin (Sigma-Aldrich, St. Louis, MO; USA) at 37°C in a fully humidified 5% CO_2_ atmosphere. Cell lines were routinely tested for Mycoplasma contamination using a custom nested PCR protocol. Cell line identity was confirmed by STR and/or SNP profiling.

**Extraction of total DNA and RNA, reverse transcription, and quantitative Real-Time PCR (qRT-PCR)**

Total DNA extraction was performed using the NucleoSpin Tissue mini kit (Macherey-Nagel, Düren, NRW, Germany). RNA was extracted using the NucleoSpin RNA mini kit (Macherey-Nagel, Düren, NRW, Germany), which included a 15-minute DNase-treatment, and was reversely transcribed using the High-Capacity cDNA Reverse Transcription Kit (Applied Biosystems, Foster City, CA, USA). qRT-PCRs were performed in a final volume of 15 µl using SYBR Select Master Mix (Applied Biosystems, Foster City, CA, USA) or TaqMan Fast Advanced Master Mix (Thermo Fisher, Waltham, MA, USA). All primer sequences used for qRT-PCR are listed in **Supplementary Table S1**. Cycling conditions were as follows: 50°C for 2 minutes (UNG/UDG incubation), 95°C for 2 minutes (polymerase activation), then 40 cycles at 95°C for 15 seconds (denaturation) and 60°C for 1 minute (annealing, elongation, and detection).

**Cloning, plasmid design, and lentiviral transduction**

Cloning and plasmid design were performed in collaboration with the Cellular Tools Core Facility at the DKFZ. A pENTR223- *SMARCB1* entry vector was used to recombine the *SMARCB1* coding sequence into the doxycycline-inducible expression vector rwpSMART-Tre3G-GW-mCMV-Puro-2A-TetON3G (CellularTools CF) via Gateway technology (ThermoFisher, Waltham, MA, USA). Correct insertion into the plasmids was verified by Sanger-sequencing and agarose gel electrophoresis. Verified, endotoxin-free plasmids were then used for lentiviral transduction. Briefly, HEK293FT cells (ThermoFisher, Waltham, MA, USA) were co-transfected with the lentiviral constructs (rwpSMART-Tre3G-GW-mCMV-Puro-2A-TetON3G with the *SMARCB1* ORF containing a stop codon) and second-generation viral packaging plasmids VSV.G (Addgene, Watertown, MA, USA, #14888) and psPAX2 (Addgene, Watertown, MA, USA, #12260). 48 hours after transfection, the supernatant containing viral particles was collected, cleared by centrifugation (5 minutes at 500 g) and passed through a 0.45 μm filter (Merck, Darmstadt, Hessen, Germany) to remove remaining cellular debris. EpS cells were transduced with lentiviral particles at 80% confluency in the presence of 10 μg/ml polybrene (Merck, Darmstadt, Hessen, Germany) for 24 hours. Only Eps cell lines with no detectable SMARCB1 protein expression and characteristics of homozygous SMARCB1-deletion (FU-EPS-1; HS-ES-1, -2M, -2R; NEPS; VA-ES-BJ) were selected for viral transduction. Transduced cells were selected with 1 μg/ml puromycin (Invivogen, San Diego, CA, USA) for ~1 week. Stably transduced isogenic cell lines were derived from single colonies and characterized for mRNA expression by TaqMan qRT-PCR.

Dox levels for *SMARCB1* re-expression cell lines were titrated to quasi-physiological levels. TaqMan qPCR was performed with EwS cell lines (A-673, MHH-ES1, SK-N-MC, TC-106, and TC-71), which do not show perturbation of the *SMARCB1* gene, alongside *RPLP0* as a known housekeeping gene in EwS. Expression levels were cross-referenced with nTPM values obtained from the Human Protein Atlas (HPA; [www.proteinatlas.org](http://www.proteinatlas.org)) [1]. Across EwS cell lines, a relatively narrow variance in *SMARCB1* expression was observed, representing the lower end of the range of *SMARCB1* expression found in non-cancerous cell lines obtained from the HPA (<https://www.proteinatlas.org/ENSG00000099956-SMARCB1/cell+line#non-cancerous>). Since the cell of origin is unknown for EpS, *SMARCB1* expression in these cell lines was adjusted to these levels instead.

**Western blot**

Western blots were performed as previously described [2]. For preparation of protein lysates, 4–5×10^5^ cells (depending on the cell line) were seeded per well in 6-well plates to reach 80% confluency after 72 hours (~1×10^6^ cells). Thereafter, the medium was removed, and cells were washed with 1 ml of PBS and lysed by adding 100 µl of RIPA buffer (Serva electrophoresis, Heidelberg, BW, Germany), supplemented with cOmplete, Mini, EDTA-free Protease Inhibitor Cocktail and PhosStop (Roche, Basel, Switzerland). Antibodies were used to detect BRG1 (Abcam, Cambridge, UK, ab110641, 1:10,000), SMARCB1 (Cell Signaling, Danvers, MA, USA, #91735, 1:1,000), Lamin A/C (Santa Cruz, Dallas, TX, USA, sc376248, 1:100), or GRP94 (Cell Signaling, Danvers, MA, USA, #20292, 1:1,000) by reaction with a secondary HRP-conjugated, monoclonal murine anti-rabbit antibody (sc-2357, 1:2,000, Santa Cruz, Dallas, TX, USA) or HRP-conjugated m-IgGκ BP-HRP (sc-516102, 1:1,000, Santa Cruz, Dallas, TX, USA).

**Clonogenic growth assays**

For clonogenic growth assays, *SMARCB1* re-expressing EpS cells and respective controls were seeded in triplicate wells at low density (5×10^2^ cells per well) in 12-well plates and grown for 10–14 days (depending on the cell line) with or without treatment (BRM014 (Adooq, Cologne, Germany), DOX (Beladox, Bela-pharm, Oldenburg, Germany) or vehicle (DMSO, Sigma-Aldrich, St. Louis, MO; USA) every 48 hours). Thereafter, colonies were stained with crystal violet (Sigma-Aldrich, St. Louis, MO; USA) for visualization of clonogenicity using ImageJ. Data were then fitted with a three-parameter logistic model by non-linear regression, and the IC_50_ was calculated.

**Cell cycle analysis by flow cytometry**

Prior to analysis, cells were harvested after 96 hours treatment with DOX (refreshed every 48 hours) by centrifugation at 1,200 rpm for 4 minutes, then washed with PBS and spun down at 2,000 rpm for 4 minutes to collect cells. Excess medium was carefully aspirated without disturbing the cell pellet, leaving about 100 µl in flask. To fix the cells, the pellet was vortexed while adding 1 ml of ice-cold EtOH (70%) dropwise and stored at 4 °C until analysis. For flow cytometric cell cycle analysis via propidium iodide (PI) staining, fixed samples were centrifuged at 2,000 rpm for 10 minutes, then washed twice with cold PBS. Excess medium was aspirated after every washing step. Each sample was resuspended in 500 µl of PI staining buffer (470 µl FACS staining buffer (BioLegend, CA, USA), 25 µl PI solution (BioLegend, CA, USA), and 5 µl RNase A (VWR International, PA, USA) and incubated in the dark at room temperature. Analysis using a BD FACS Canto II system was performed within 3 hours after staining, in slow/medium flow mode. *.fcs output files were gated by FSC-A; SSC-A to define the population by size and granulation, and by FSC-A; FSC-H/W to rule out doublets. Single-cell populations were then analyzed by PI stain in FlowJo (v10) to determine the counts for each cell cycle phase (G1/G0, S, G2/M).

**Transcriptome analyses**

To assess the effect of *SMARCB1* rescue on gene expression in EpS cells, microarray analyses were performed. For this, 5×10^4^ cells per well were seeded in 6-well plates and treated with 0.1 μg/ml DOX for 96 hours (DOX-refreshment after 48 hours). Thereafter, total RNA was extracted using the NucleoSpin RNA mini kit (Macherey-Nagel, Düren, NRW, Germany), and RNA quality was assessed with a TapeStation 4150 system using an RNA Screentape (Agilent, Santa Clara, CA, USA). All samples had an RNA integrity number (RINe) > 9 and were hybridized to Human Affymetrix Clariom D microarrays (Thermo Fisher, Waltham, MA, USA). Gene expression data were quantile normalized with Transcriptome Analysis Console (v4.0.2; Thermo Fisher, Waltham, MA, USA) using the SST-RMA algorithm as previously described [3]. Data annotation was performed using the Affymetrix library for the Clariom D Array (version 2, *Homo sapiens*) on the gene level. Differentially expressed genes (DEGs) with consistent and significant fold changes (FCs) across cell lines were identified as follows: i) Normalized gene expression signals were log_2_ transformed; ii) to avoid false discovery artifacts due to detection of minimally expressed genes, all genes with an equal or lower expression value than that observed for *SMARCB1* in the control cell lines (all with homozygous *SMARCB1* deletion) across all cell lines and conditions were excluded. The FCs of the empty vector samples and all *SMARCB1* re-expressing EpS cell lines after treatment (DOX or BRM014) were calculated for each cell line separately. Then, the FCs in the *SMARCB1* re-expressing samples were normalized to that of the empty vector cells (for DOX treatment). Finally, resulting FCs were filtered for genes equally down- or up-regulated across cell lines, and then averaged to obtain the mean FC per gene. DEGs were determined as having a |log_2_FC| > 0.5.

**Gene-set enrichment analysis (GSEA) and WGCNA**

To identify enriched gene-sets, genes were ranked by their expression FC between treatment and control groups, then additionally filtered for unidirectionally aligned regulation in all cell lines for pooled analyses (i.e. up-/downregulated in all cell lines upon DOX treatment). GSEA (multilevel) was performed using the FGSEA R package (v3.6.3) based on Gene Ontology (GO) biological processes and cell signature terms from MSigDB (c5.go.bp.v7.5.1.symbols.gmt and c8.all.v2023.2.Hs.symbols.gmt) [4]. GO terms were filtered for statistical significance (adjusted *p-*value < 0.05) and a normalized enrichment score |NES| > 2. To construct a network, the Weighted Gene Correlation Network Analysis R package (WGCNA R) [5] was used. Briefly, a binary matrix of GO-terms × genes (where 1 indicates the gene is present in the GO term and 0 indicates it is not) was created. Then, Jaccard’s distance for all possible pairs was computed to create a symmetric GO adjacency matrix. Clusters of similar GO terms were identified using the dynamicTreeCut algorithm, and the top 20% of the highest edges were selected for visualization. The most meaningful among the highest scoring nodes in each cluster was determined as the cluster label. The obtained network and nodes files were processed using Cytoscape (v 3.8.0) for network design and visualization as previously described [6]. GOChord visualization for the most highly enriched gene sets/cell signatures was performed with the GOPlot R package [7]. Only genes contained within leadingEdge analysis of plotted cell signature gene sets were shown.

**Protein sample preparation and immunoprecipitation followed by mass spectrometry**

Nuclear and whole-cell extracts were prepared from 1.5×10^7^ cells per condition, with or without DOX treatment for 4 days, using the Nuclear Extract Kit (Active Motif, Carlsbad, CA; USA). Co-immunoprecipitation was performed on the nuclear fraction using the Dynabeads Co-Immunoprecipitation Kit (Thermo Fisher, Waltham, MA, USA) coupled with the anti-BRG1 antibody ab110641 (rabbit monoclonal, Abcam), according to the manufacturer’s manual. Mass spectrometry was performed at the DKFZ Core Facility for Genomics and Proteomics as follows:

Proteins (5 µg) were run 0.5 cm into an SDS-PAGE, and the entire piece was excised and digested using trypsin, as described by Shevchenko et al. [8], with adaptations for a DigestPro MSi robotic system (INTAVIS Bioanalytical Instruments AG). LC-MS/MS analysis was carried out on a Vanquish Neo UPLC (Thermo Fisher, Waltham, MA, USA) directly connected to an Orbitrap Exploris 480 mass spectrometer over a 90-minute run. Peptides were online desalted on a trapping cartridge (Acclaim PepMap300 C18, 5 µm, 300 Å wide pore; Thermo Fisher, Waltham, MA, USA) using the metering device at a flow rate of 30 µl/minute for a total loading volume of 60 µl. The analytical multistep gradient (300 nl/minute) was performed using a nanoEase MZ Peptide analytical column (300 Å, 1.7 µm, 75 µm x 200 mm, Waters, Milford, MA; USA) with solvent A (0.1% formic acid in water) and solvent B (0.1% formic acid in acetonitrile). For 72 minutes, the concentration of solvent B was linearly ramped from 4% to 30%, followed by a quick ramp to 80%. After 4 minutes at 80%, the concentration of B was reduced to 2%, followed by a three-column volume equilibration step. Eluting peptides were analyzed using data-dependent acquisition (DDA) mode. A full scan at 60k resolution (380–1,400 m/z, 300% AGC target, 45 ms maxIT) was followed by up to 1.5 seconds of MS/MS scans. Peptide features were isolated with a window of 1.4 m/z and fragmented using 26% NCE. Fragment spectra were recorded at 15k resolution (100% AGC target, 54 ms maxIT). Dynamic exclusion was set to 30 seconds. Data analysis was performed with MaxQuant (version 2.1.4.0, ref. [9]) using an organism-specific database extracted from Uniprot.org (human reference database with one protein sequence per gene, containing 20,597 unique entries, February 9, 2024). Default settings were used with the following adaptions. Match between runs (MBR) was enabled to transfer peptide identifications across raw files based on accurate retention time and m/z. Fractions were set to enable MBR only within replicates. Separate parameter groups were assigned for cell line and proteome fraction or IP. Separate label-free quantification (LFQ) per parameter group was enabled. Quantification was performed using the MaxLFQ algorithm [10], requiring a minimum of 2 quantified peptides per protein. Additionally, iBAQ-values [11] were generated.

LFQ results were normalized to the column sum per experimental subgroup, and missing values imputed using slsa (for partially observed values) and det quantile (quantile 2.5, factor 1, for values missing in the entire collection) before hypothesis testing in Prostar [12]. Resulting FCs were filtered for genes/proteins consistently down- or up-regulated across cell lines, and then averaged to calculate the mean FC per gene. GSEA followed by WGCNA was performed on prefiltered gene/protein lists as detailed in the GSEA/WGCNA section. Visualized gene sets were filtered for |NES| > 2.5 (downregulated upon *SMARCB1* re-expression in BRG1 Co-IP) or |NES| > 2 (all other conditions) to maintain legibility.

Differentially enriched proteins (DEP) were defined as having a |log_2_FC| > 1 and adjusted *p*-value < 0.05. GSEA were performed on DEP from the BRG1 Co-IP (+/– re-expression of *SMARCB1*) condition using the “Protein-protein interaction (PPI) hub protein” sets within the Enrichr tool [13]. GOChord visualization was performed with the GOPlot R package [7].

**Assay for transposase-accessible chromatin followed by next-generation sequencing (ATAC-Seq)**

Nuclei isolation and sample preparation were performed using the ATAC-Seq kit and the 24 UDI for Tagmented libraries - Set I (Diagenode, Seraing, Belgium) with 5×10^4^ cells per condition after treatment with DOX or BRM014 (1 µmol/L) for 4 days, according to the manufacturer’s protocol. In brief, cells were treated, harvested, and lysed. Nuclei were then isolated, and DNA was tagmented. Library amplification was performed using unique dual index primers. Size selection was performed on the libraries with AMPure XP beads (Beckman, Brea, CA, USA). Finally, quality control was performed with the Qubit DNA Assay and the Agilent TapeStation 4150 system with a D5000 Screentape.

An equimolar multiplex was created from purified libraries and adjusted to 10 nmol/L. Sequencing was performed on a NovaSeq 6000 Paired-End 100bp S4 chip by the DKFZ NSG Core facility. Using the DKFZ galaxy platform, the sequencing reads were pre-processed with TrimGalore! (v0.6.7) to remove adapter sequences, then mapped with bowtie2 (v2.5.1) to the hg19 reference genome. Post-processing was conducted using Filter BAM (v2.5.2) to filter out improperly paired reads, reads with a mapping quality less than 30, and/or reads mapping to the mitochondrial chromosome. Duplicate reads were removed using MarkDuplicates (v3.1.1.0). Processed reads were visualized in IGV using bamCoverage (v3.5.4). Peaks were called using MACS2 (v2.2.9.1) on the BAM datasets. Differential peak enrichment analysis between treatment conditions and control was performed using the DiffBind R package [14]. Motif analysis on differential peaks was performed using the MEME suite (MEME-ChIP and SEA) on genomic sequences extracted from peak files. Differential motif analysis was performed with SEA using the respective other condition as background sequences (e.g., BRM014 associated sequences as background for DOX/*SMARCB1* re-expression associated sequences and vice versa). Differential peaks were filtered by fold enrichment (> 2 for BRM014 treatment pooled analysis, > 3 for DOX treatment subtype analysis) and analyzed using the GREAT (Ver 4.0.4) tool developed by Stanford University [15,16]. Each gene was assigned a basal regulatory domain 5 kb upstream and 1 kb downstream of its TTS, extended distally by a maximum of 1 Mb in each direction without overlapping with the basal domain of the nearest gene, and regions were associated with all their overlapping genes.

Correlation matrices were computed using the ComputeMatrix tool, with the whole genome summarized into 10 kb bins or DiffBind site bins, respectively. The matrices were analyzed using Graphpad Prism for Pearson correlation.

**Chromatin immunoprecipitation followed by DNA sequencing (ChIP-Seq)**

H3K4me3, H3K27ac, and H3K27me3 chromatin immunoprecipitation (ChIP) was performed using the iDeal ChIP-Seq kit for histones or the iDeal ChIP-Seq kit for transcription factors (Diagenode, Seraing, Belgium) using the following ChIP-Seq grade antibodies: C15410003 (rabbit polyclonal, Diagenode, Seraing, Belgium) for H3K4me3, ab4729 (rabbit polyclonal, Abcam) for H3K27ac, C15410069 (rabbit polyclonal, Diagenode) for H3K27me3, #91735 (rabbit monoclonal, Cell Signaling) for BAF47, and ab110641 (rabbit monoclonal, Abcam) for BRG1. 2×10^7^ cells were cross-linked with 1% formaldehyde for 10 minutes and quenched with 125 mmol/L glycine (final concentration) for 10 minutes at room temperature. Chromatin was isolated by the addition of lysis buffer, and lysates were sonicated to obtain chromatin fragments averaging ∼300 bp in length. ChIP was performed with chromatin of 1 million cells for histone marks and 4 million cells for transcription factors (TFs). An equivalent of 1% of the chromatin used for TFs was reserved to quantify the input. ChIP was performed overnight at 4 °C on a rotating wheel with 1.4 μg of antibody for H3K4me3, 1 μg for H3K27ac, 2.9 μg for H3K27me3, 1.4 μg for SMARCB1, and 7 μg for BRG1. After ChIP, chromatin was eluted for 30 minutes on a DiaMag rotator at room temperature in 100 µl iE1 buffer and reverse cross-linked for 4 hours at 65°C with shaking in iE2 buffer. DNA was precipitated and purified using magnetic beads with the IPure v2 kit (Diagenode). Before sequencing, ChIP efficiency was validated by qPCR for each antibody on specific genomic regions using powerSYBR Green Master Mix (Applied Biosystems). Efficiency was compared for each primer pair to the input DNA. Primers are listed in **Supplementary Table S1.**

Library preparation from ChIP-fragments was performed using the MicroPlex Library Preparation Kit v3 and the ‘24 UDI for MicroPlex v3 - Set I’ (Diagenode, Seraing, Belgium). A 10 µl sample containing 50 pg of precipitated DNA was used for library preparation according to the manufacturer’s protocol for template preparation and library amplification. Amplified intermediate libraries were quantified using the Agilent TapeStation 4150 system on D5000 HS Screentape to evaluate fragment distribution, unincorporated adapters, and DNA concentration. If yields were inadequate, re-amplification was performed for 2–3 cycles under the same conditions as the library amplification protocol.

Completed libraries were purified using AMPure® XP beads in a 1:0.9 volume ratio of sample to beads and eluted in 15 µl of low TE buffer. Libraries were then quantified using the TapeStation 4150 system on D5000 HS Screentape.

An equimolar multiplex of purified libraries, adjusted to 10 nmol/L, was sequenced on a NovaSeq 6000 Paired-End 50bp SP chip at the DKFZ NSG Core facility. Sequencing reads were mapped using BWA for medium and long reads (>150 bp) against the hg19 reference genome (v7.17.1) and post-processed using NGS tools, including SAMtools and Filter SAM or BAM (v1.1.1) for reads with mapping quality less than 20. Correlation among different targets was assessed using NGS: DeepTools, multiBamSummary (v3.3.2.0.0). Processed reads were visualized in IGV using bamCoverage (v3.3.2.0.0). Peaks were called on the BAM datasets using MACS2 (v2.2.7.1). Motif analysis was performed using the MEME suite (MEME-ChIP and SEA) on genomic sequences extracted from peak files. Differential peak enrichment analysis was performed with the DiffBind R package [14]. Resulting BED files were analyzed using the GREAT (Ver 4.0.4) tool developed by Stanford University [15,16]. Each gene was assigned a basal regulatory domain 5 kb upstream and downstream of its TTS, extended distally by up to 1 Mb in each direction without overlapping with the basal domain of the next nearest gene. Regions were associated with all immediately overlapping genes. For analysis of promoter categories, H3K4me3 and H3K27me3 histone mark peaks were associated with gene TSS within 2 kb upstream or downstream of the peaks using GREAT and overlapped with BRG1 peak-associated genes.

**Mouse xenograft experiments**

For subcutaneous xenograft experiments, 2.5×10^6^ wild-type or pre-transduced VA-ES-BJ or NEPS EpS cells, suspended in a 1:1 mix of PBS and Geltrex (LDEV-Free, hESC-Qualified, Reduced Growth Factor Basement Membrane Matrix-5 mL. A1413302, Gibco/LifeTechnologies, Thermo Fisher, Waltham, MA, USA), were subcutaneously injected into the flank of NSG mice. Tumor growth was measured three times a week using a caliper. Tumor volumes were calculated using the following formula: V = L × W^2^ / 2, where V is the tumor volume, L is the largest diameter and W is the smallest diameter. When tumors reached an average volume of 60 mm^3^, mice were randomized in two groups. One group was treated with 2 mg/ml DOX (Beladox, Bela-pharm, Germany) dissolved in drinking water containing 5% sucrose (Sigma-Aldrich) to induce *in vivo* re-expression (DOX (+)), while the other group received only 5% sucrose (control, DOX (−)). Before the tumors in control groups exceeded a maximum length of 15 mm in any dimension or an average volume of 1,500 mm^3^, all mice were sacrificed by cervical dislocation. Humane endpoints were determined as follows: ulcerated tumors, loss of 20% body weight, constant curved or crouched body posture, bloody diarrhea or rectal prolapse, abnormal breathing, severe dehydration, visible abdominal distention, obese body condition scores (BCS), motor irregularities, aggressiveness as a sign of pain, self-mutilation, apathy, self-isolation, maximum observation period of 12 months, and/or invasive tumor growth with fluid leakage (e.g., blood, serous fluid), functional impairment, disability, or pain. Animal experiments were conducted with approval from the government of North Baden (NTP-ID: 00029631-1-6) and in accordance with the 3R principle of animal experiments (replacement, reduction, and refinement), ARRIVE guidelines, recommendations of the European Community (86/609/EEC), and UKCCCR (guidelines for the welfare and use of animals in cancer research).

For the treatment of pre-transduced xenografts, BRM014 (solubilized in 5% DMSO, 10% Kolliphor, and 85% (10% beta-CD in sterile water)) was administered via intraperitoneal (i.p.) injection at 20 mg/kg, 5 days a week, for a maximum of 2 weeks [17,18]. After extraction of the tumors, a small fraction of each tumor was snap-frozen in liquid nitrogen for preservation, while the remaining tumor tissue was fixed in 4%-formalin and embedded in paraffin for immunohistological analysis. The statistical significance of longitudinal growth was calculated with the TumGrowth online tool by Enot et al. [19].

**Histology**

For immunohistochemistry (IHC), 4-μm sections were cut and stained with H&E (#T.865.3, Mayer, Roth). Slides were scanned using a Nanozoomer-SQ Digital Slide Scanner (Hamamatsu Photonics K.K., Hamamatsu City, Shizuoka, Japan) and visualized using the NDP.view2 image viewing software (Hamamatsu Photonics K.K., Hamamatsu City, Shizuoka, Japan). Necrosis percentage and mitosis counts were scored and averaged across 10 High-Power-Fields (HPF) at 40× magnification, then statistically analyzed (treatment vs. control) using a one-sided, unpaired t-test.

**Cell viability assays**

A total of 2~3×10³ EpS cells were seeded in 90 µl of medium per well in a 96-well plate. After 24 hours, drugs were added to a final concentration, with 0.05% dimethyl sulfoxide (DMSO) present in all conditions. 48 hours after the addition of the test drugs, cell viability was assessed using a Resazurin-based readout system [20]. Relative fluorescence units from treated wells were background corrected and normalized to vehicle controls.

**Drug synergy assays**

Drug synergy assays were performed and analyzed similarly to clonogenic growth assays (see above) with the following modifications: 12-well plates were instead incubated with a drug matrix (DOX × BRM014) at 0, 100 and 1,000 ng/ml (approximately 0, 225 and 2,250 nmol/L) for DOX and 0, 1, 10, 100 nmol/L for BRM014. All wells were equilibrated to the same DMSO vehicle concentrations (0.001%). Drug addition was performed every 48 hours. For determination of cell viability, colony area was normalized to the control well (0 DOX, 0 BRM014). Drug synergy analysis was performed with the SynergyFinder tool (v3.0) using the Bliss algorithm [21].

**Statistical analysis**

Data was analyzed using GraphPad PRISM 9 (GraphPad Software, San Diego, CA, USA). Where not otherwise specified, the statistical significance of differences between two experimental groups was tested using the two-tailed Wilcoxon Rank Sum / Mann-Whitney test. *P*-values < 0.05 were considered statistically significant. Data visualization was performed in Graphpad PRISM 9.

**Data availability**

Original Affymetrix transcriptome profiling data have been deposited in the Gene Expression Omnibus (GEO) under the accession code GSE276634. Proteomics data have been deposited in the Proteomics Identifications Database (PRIDE) under PXD053945. ATAC-Seq and ChIP-Seq data have been deposited in GEO under the accession series codes GSE281434 and GSE281436.

**Supplementary references**

1. Karlsson M, Zhang C, Méar L, Zhong W, Digre A, Katona B, et al. A single-cell type transcriptomics map of human tissues. Sci Adv. 2021 Jul;7(31):eabh2169.

2. Marchetto A, Romero-Pérez L. Western Blot Analysis in Ewing Sarcoma. Methods Mol Biol. 2021;2226:15-25.

3. Marchetto A, Ohmura S, Orth MF, Knott MML, Colombo MV, Arrigoni C, et al. Oncogenic hijacking of a developmental transcription factor evokes vulnerability toward oxidative stress in Ewing sarcoma. Nat Commun. 2020 May 15;11(1):2423.

4. Subramanian A, Tamayo P, Mootha VK, Mukherjee S, Ebert BL, Gillette MA, et al. Gene set enrichment analysis: A knowledge-based approach for interpreting genome-wide expression profiles. Proceedings of the National Academy of Sciences. 2005 Oct 25;102(43):15545-50.

5. Langfelder P, Horvath S. WGCNA: an R package for weighted correlation network analysis. BMC Bioinformatics. 2008 Dec 29;9(1):559.

6. Waszak SM, Robinson GW, Gudenas BL, Smith KS, Forget A, Kojic M, et al. Germline Elongator mutations in Sonic Hedgehog medulloblastoma. Nature. 2020 Apr;580(7803):396-401.

7. Walter W, Sánchez-Cabo F, Ricote M. GOplot: an R package for visually combining expression data with functional analysis. Bioinformatics. 2015 Sep 1;31(17):2912-4.

[8. Shevchenko A, Tomas H, Havli J, Olsen JV, Mann M. In-gel digestion for mass spectrometric characterization of proteins and proteomes. Nat Protoc. 2006 Dec;1(6):2856-60.

9. Tyanova S, Temu T, Cox J. The MaxQuant computational platform for mass spectrometry-based shotgun proteomics. Nat Protoc. 2016 Dec;11(12):2301-19.

10. Cox J, Hein MY, Luber CA, Paron I, Nagaraj N, Mann M. Accurate Proteome-wide Label-free Quantification by Delayed Normalization and Maximal Peptide Ratio Extraction, Termed MaxLFQ. Mol Cell Proteomics. 2014 Sep;13(9):2513-26.

11. Schwanhäusser B, Busse D, Li N, Dittmar G, Schuchhardt J, Wolf J, et al. Global quantification of mammalian gene expression control. Nature. 2011 May;473(7347):337-42.

12. Wieczorek S, Combes F, Lazar C, Giai Gianetto Q, Gatto L, Dorffer A, et al. DAPAR & ProStaR: software to perform statistical analyses in quantitative discovery proteomics. Bioinformatics. 2017 Jan 1;33(1):135-6.

13. Chen EY, Tan CM, Kou Y, Duan Q, Wang Z, Meirelles GV, et al. Enrichr: interactive and collaborative HTML5 gene list enrichment analysis tool. BMC Bioinformatics. 2013 Apr 15;14(1):128.

14. Stark R, Brown G. Bioconductor. 2011 [cited 2024 Jan 24]. DiffBind. Available from: <http://bioconductor.org/packages/DiffBind/>

15. McLean CY, Bristor D, Hiller M, Clarke SL, Schaar BT, Lowe CB, et al. GREAT improves functional interpretation of cis-regulatory regions. Nat Biotechnol. 2010 May;28(5):495-501.

16. Tanigawa Y, Dyer ES, Bejerano G. WhichTF is functionally important in your open chromatin data? PLoS Comput Biol. 2022 Aug;18(8):e1010378.

17. Panditharatna E, Marques JG, Wang T, Trissal MC, Liu I, Jiang L, et al. BAF Complex Maintains Glioma Stem Cells in Pediatric H3K27M Glioma. Cancer discovery. 2022;12(12):2880-905.

18. Mo Y, Duan S, Zhang X, Hua X, Zhou H, Wei HJ, et al. Epigenome programing by H3.3K27M mutation creates a dependence of pediatric glioma on SMARCA4. Cancer Discov. 2022 Dec 2;12(12):2906-29.

19. Enot DP, Vacchelli E, Jacquelot N, Zitvogel L, Kroemer G. TumGrowth: An open-access web tool for the statistical analysis of tumor growth curves. Oncoimmunology [Internet]. 2018 [cited 2024 Jan 19];7(9). Available from: <https://www.ncbi.nlm.nih.gov/pmc/articles/PMC6140814/>

20. Musa J, Cidre-Aranaz F. Drug Screening by Resazurin Colorimetry in Ewing Sarcoma. In: Cidre-Aranaz F, G. P. Grünewald T, editors. Ewing Sarcoma : Methods and Protocols [Internet]. New York, NY: Springer US; 2021 [cited 2024 Sep 16]. p. 159–66. Available from: <https://doi.org/10.1007/978-1-0716-1020-6_12>

21. Ianevski A, Giri AK, Aittokallio T. SynergyFinder 3.0: an interactive analysis and consensus interpretation of multi-drug synergies across multiple samples. Nucleic Acids Research. 2022 Jul 5;50(W1):W739-43.

**Supplementary Figure Legends**

**
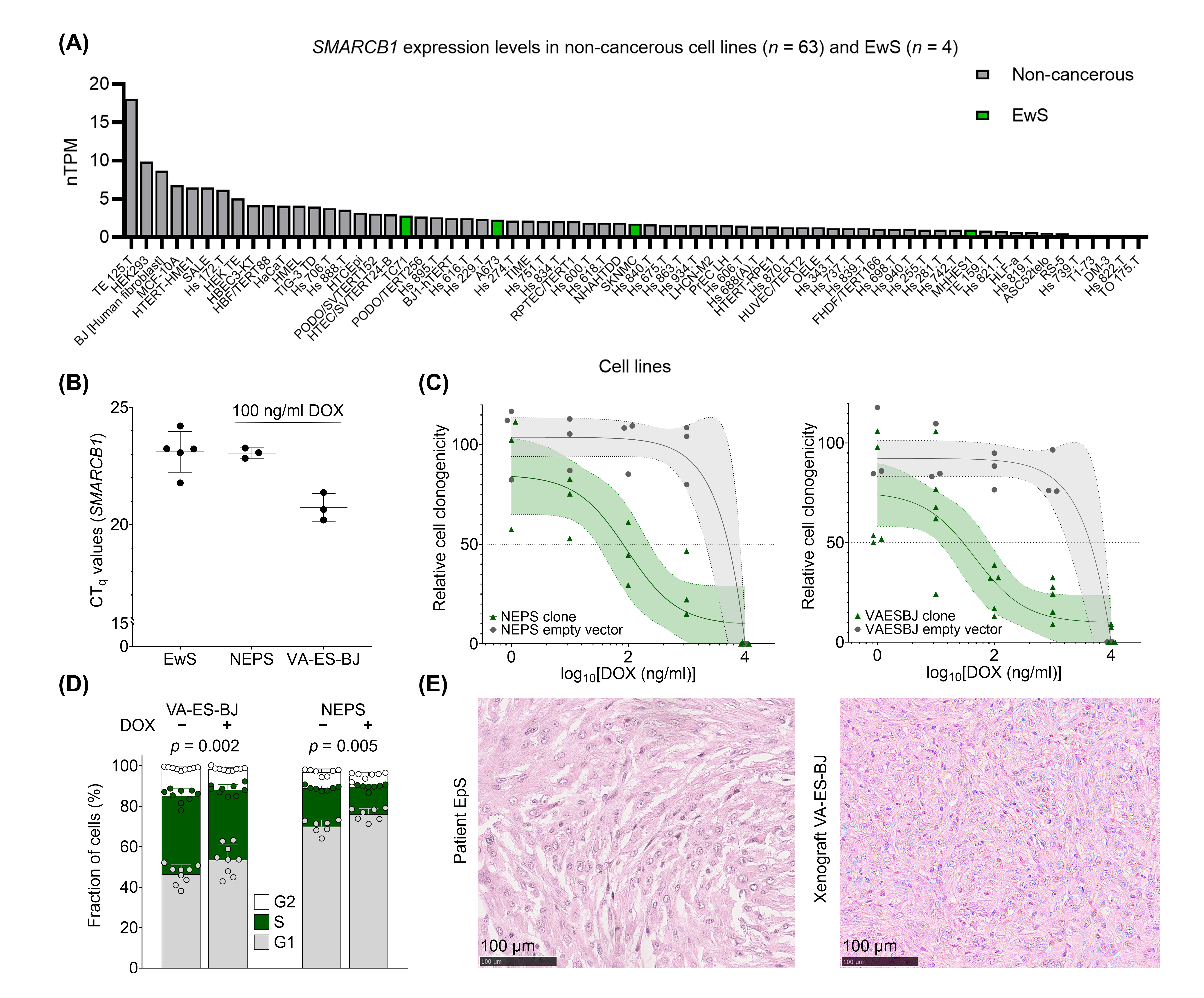
**

**Supplementary Figure S1. Functional analyses of isogenic EpS models at pseudophysiological *SMARCB1* expression levels confirm the regulation of biological processes predicted by transcriptomic analyses.**

**(A)** Expression levels (nTPM) of *SMARCB1* in non-cancerous cell lines (data source: Human Protein Atlas), showing a conserved trend for *SMARCB1* re-expression across various tissues.

**(B)** CT_q_ values of wild-type EwS cell lines (A-673, SK-N-MC, MHH-ES1, TC-106, TC-71) compared to NEPS and VA-ES-BJ after *SMARCB1* re-expression (with 100 ng/ml DOX), demonstrating a comparable expression level to most tissues shown in (A), with EwS cell lines included in both analyses.

**(C)** Clonogenicity response curves of NEPS and VA-ES-BJ clones following *SMARCB1* re-expression compared to empty vector controls at increasing DOX concentrations, showing dose-dependent titration of gene effect.

**(D)** Delayed G1/S-phase cell cycle progression upon *SMARCB1* re-expression in VA-ES-BJ and NEPS, shown using flow-cytometric PI staining.

**(E)** Comparison of HE-stained patient and xenograft tumor tissue, showing similar histologic features. Black bars in the lower-left corner measure 100 µm.

Abbreviations: nTPM, normalized transcripts per million; SMARCB1, SWI/SNF-related matrix-associated actin-dependent regulator of chromatin subfamily B member 1; EwS, Ewing Sarcoma; DOX, Doxycycline; PI, Propidium Iodide; H&E, Hematoxylin and eosin.

**
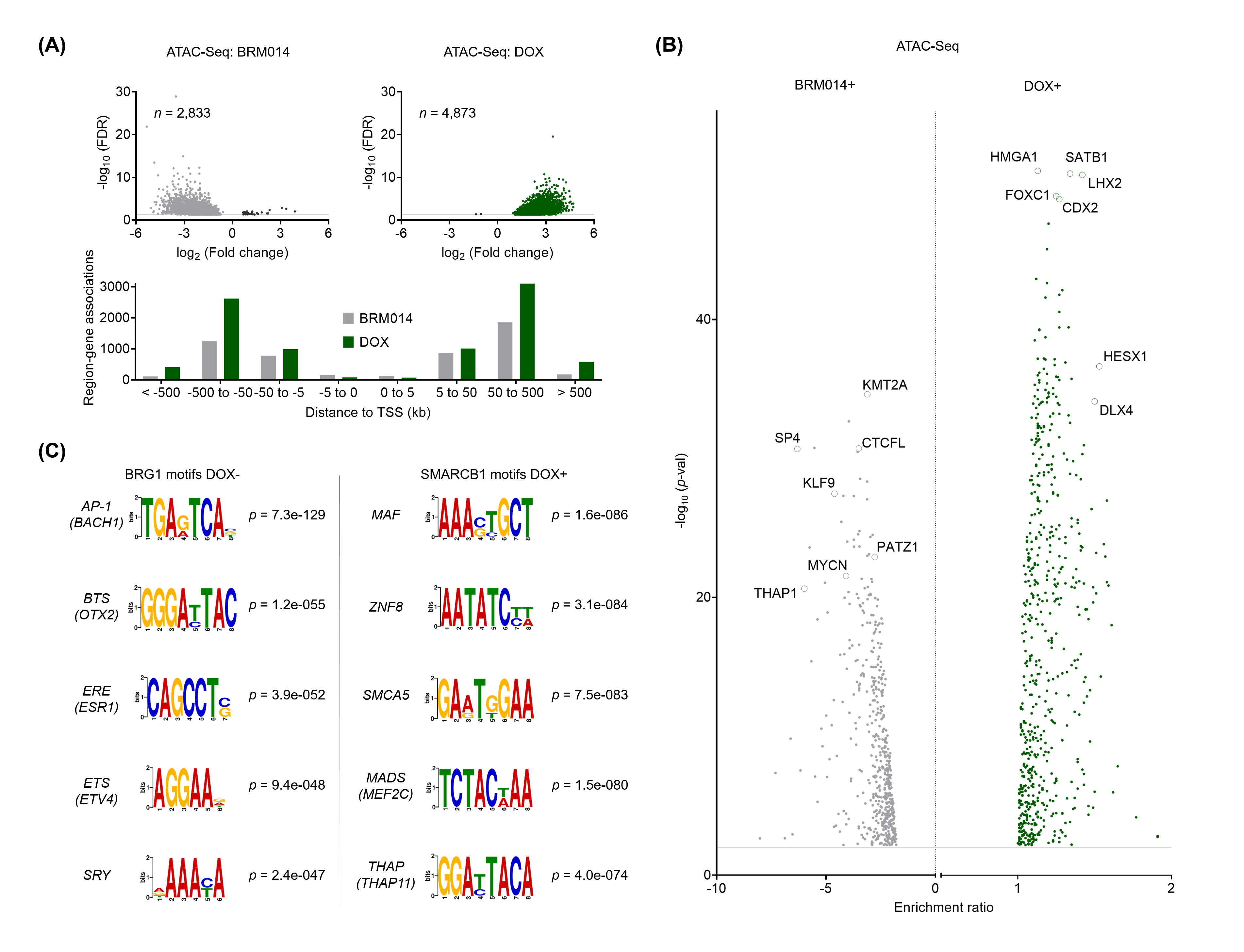
**

**Supplementary Figure S2. Differential analysis of ATAC and ChIP peaks reveals SWI/SNF motif affinity changes primarily at distal regulatory elements.**

**(A)** Volcano plots of pooled differentially enriched open chromatin regions (FDR > 0.05) after BRM014 or DOX treatment, alongside GREAT region-gene associations binned by distance to the closest gene TSS, showing modulation primarily at distal regulatory elements.

**(B)** Volcano plot of SEA demonstrating highly enriched motifs in open chromatin regions lost upon BRM014 treatment and gained upon SMARCB1 re-expression (pooled across all EpS cell lines). Each condition was computed with the other as background sequences to highlight differential motif enrichment, indicating distinct affinity for TF family motifs.

**(C)** Top enriched motifs at BRG1 (DOX-) and INI-1 (DOX+) occupied loci in VA-ES-BJ.

Abbreviations: DOX, Doxycycline; GREAT, Genomic Regions Enrichment of Annotations Tool; TSS, Transcription start site; SEA, Simple motif Enrichment Analyses; SMARCB1, SWI/SNF-related matrix-associated actin-dependent regulator of chromatin subfamily B member 1; EpS, Epithelioid Sarcoma; TF, Transcription factor.


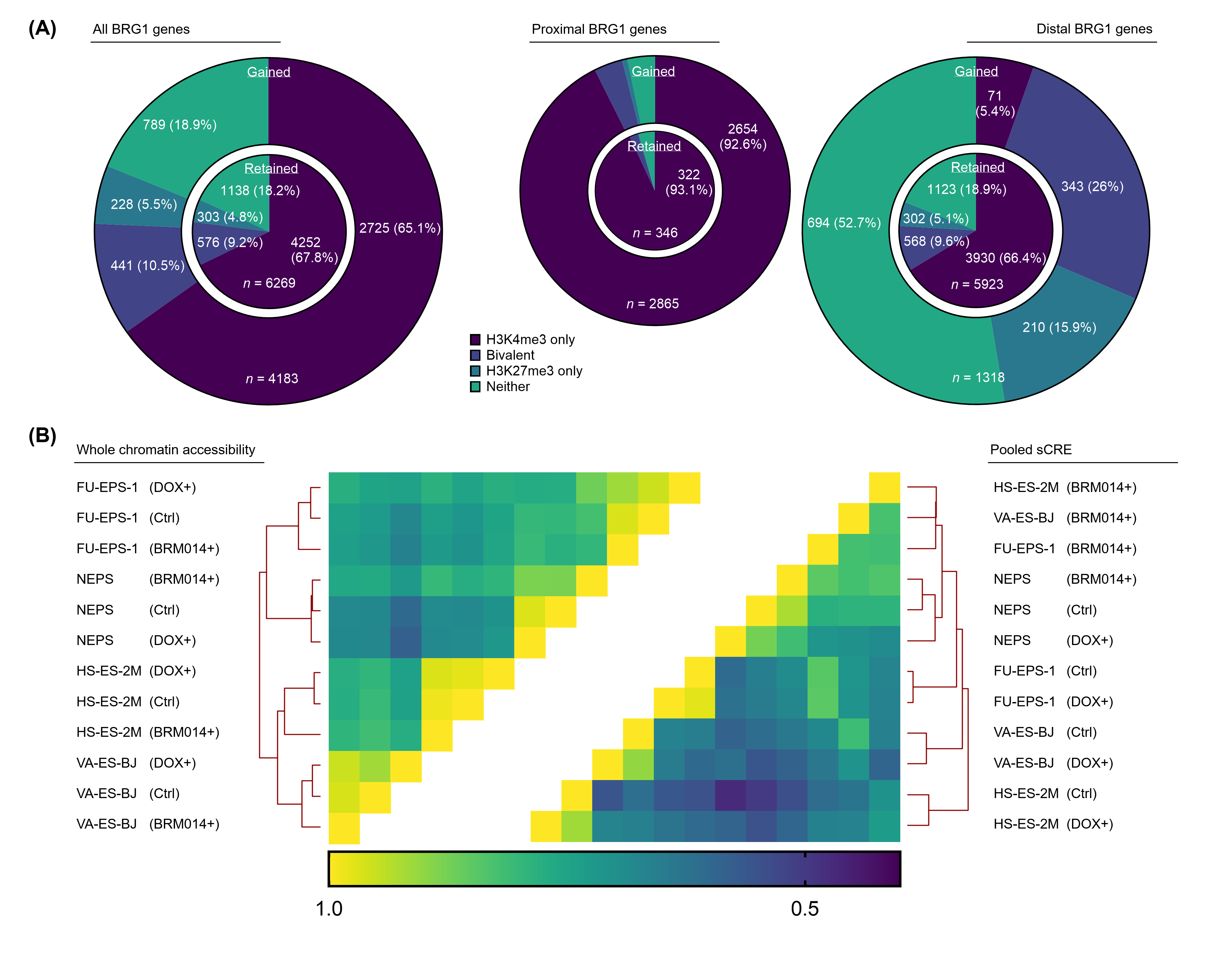


**Supplementary Figure S3.** **ATAC-Seq and ChIP-Seq reveal preferential regulation of promotor histone marks at BRG1-peak-distant genes and conserved regulation at rSWI/SNF sites.**

**(A)** Distribution of promoter categories of all, proximal (≤ 2kb from TSS), or distal (up to 1 Mb) retained (inner core) or gained (outer ring) GREAT-associated BRG1 genes upon SMARCB1 re-expression. The data highlight an increase in bivalent promoter occupancy at distal BRG1 sites gained upon SMARCB1 re-expression.

**(B)** Clustered Pearson correlation matrices of EpS cell line whole and differentially accessible chromatin regions, showing conserved modulation at sCRE sites upon rSWI/SNF inhibition.

Abbreviations: TSS, Transcription start site; GREAT, Genomic Regions Enrichment of Annotations Tool; BRG1, Brahma-Related Gene 1; SMARCB1, SWI/SNF-related matrix-associated actin-dependent regulator of chromatin subfamily B member 1; EpS, Epithelioid Sarcoma; sCRE, SWI/SNF-specific cis-regulatory elements; (r)SWI/SNF, (residual) SWItch/Sucrose Non-Fermentable.


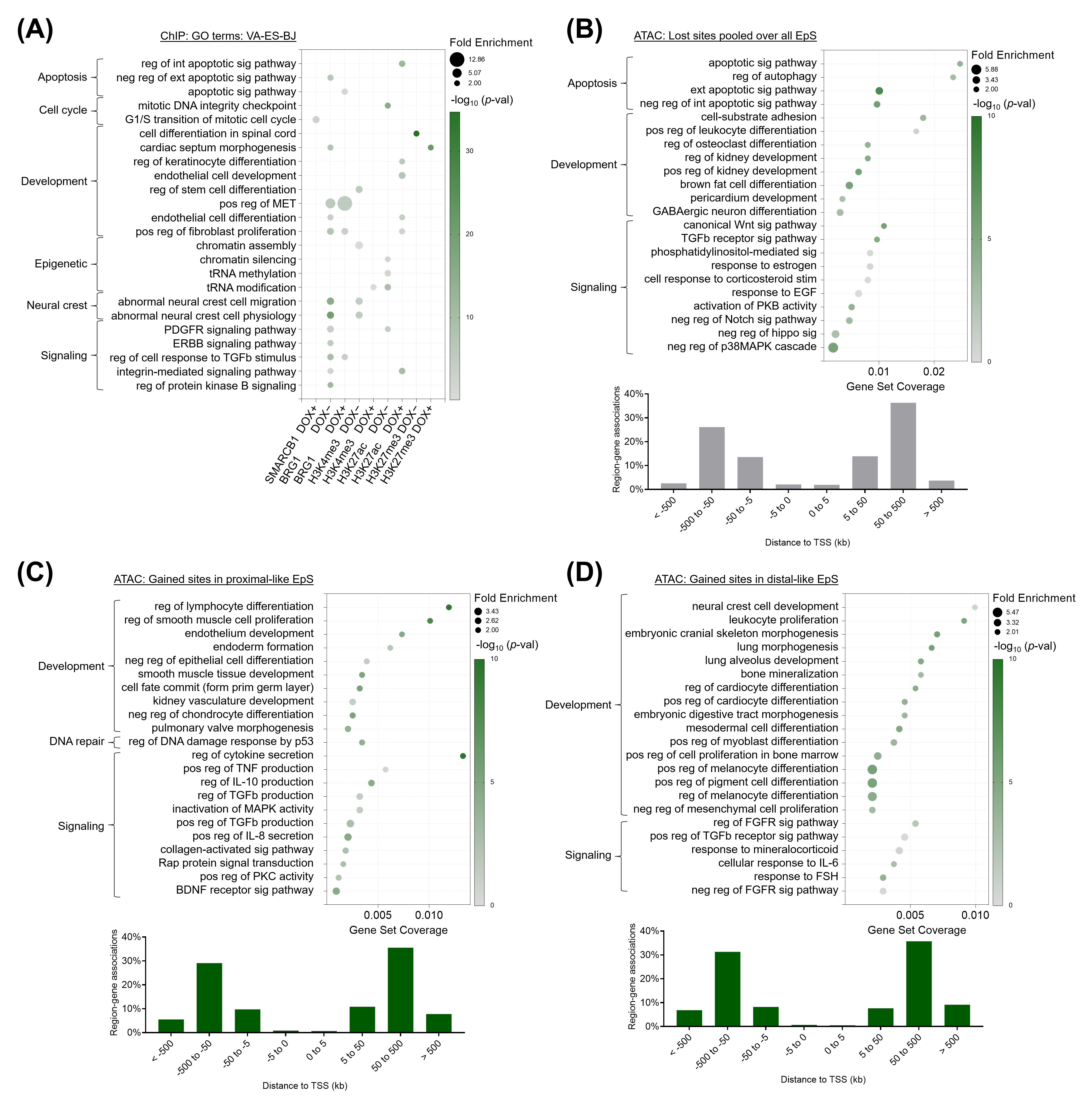
**Supplementary Figure S4. Differential analysis of ChIP-Seq and ATAC-Seq peaks yields insight into the pleiotropic, subtype-specific biological processes downstream of SWI/SNF regulation.**

**(A)** DiffBind GREAT analysis of differentially regulated SMARCB1 and BRG1 ChIP sites as well as histone marks upon re-expression of *SMARCB1*. Similar gene ontology terms were summarized.

**(B)** DiffBind GREAT analysis of sites of lost chromatin accessibility upon treatment with BRM014 alongside GREAT region-gene associations binned by distance to the closest gene TSS.

**(C-D)** DiffBind GREAT analysis of sites of gained chromatin accessibility upon re-expression of *SMARCB1* in (C) proximal- and (D) distal-like EpS cell lines, alongside GREAT region-gene associations binned by distance to the closest gene TSS.

Abbreviations: Neg, Negative; Pos, Positive; Reg, Regulation; Sig, Signaling; Ext, Extrinsic; Int, Intrinsic; Stim, Stimulation; Form, Formation; Prim, Primary; GREAT, Genomic Regions Enrichment of Annotations Tool; SMARCB1, SWI/SNF-related matrix-associated actin-dependent regulator of chromatin subfamily B member 1; BRG1, Brahma-Related Gene 1; ChIP-Seq, Chromatin immunoprecipitation followed by DNA-sequencing; TSS, Transcription start site; EpS, Epithelioid Sarcoma.

**
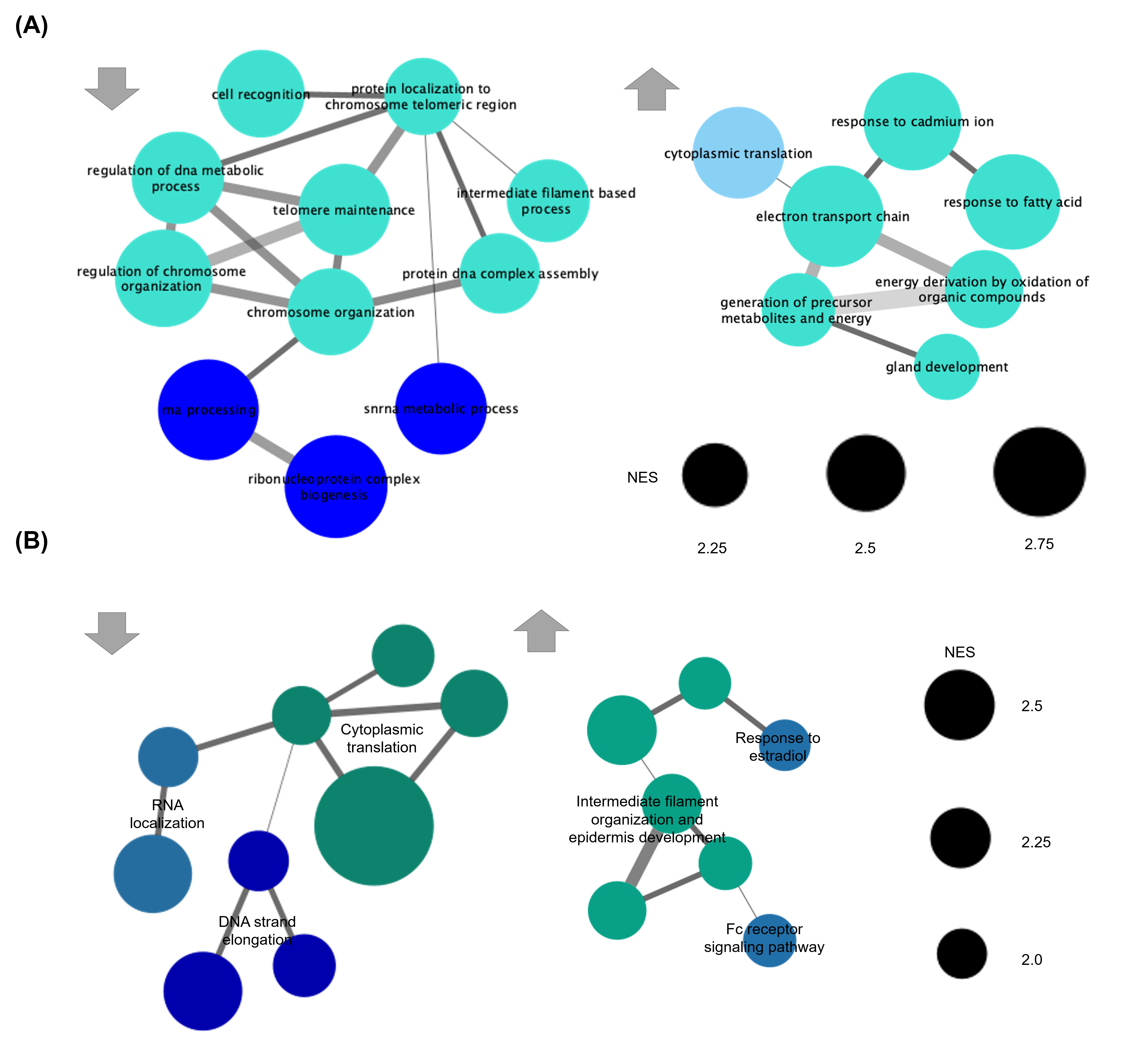
**

**Supplementary Figure S5.** **Interactomic and proteomic data support significant *SMARCB1*-associated regulation of DNA metabolism.**

GSEA-based network analysis of upregulated and downregulated biological process sets upon SMARCB1 re-expression.

**(A)** From shared regulated proteins in VA-ES-BJ and NEPS after Co-IP against BRG1 (*n* = 4 biological replicates per cell line).

**(B)** From shared regulated global proteome in VA-ES-BJ and NEPS (*n* = 4 biological replicates per cell line).

Abbreviations: GSEA, Gene set enrichment analysis; SMARCB1, SWI/SNF-related matrix-associated actin-dependent regulator of chromatin subfamily B member 1; Co-IP, Co-immunoprecipitation; BRG1, Brahma-Related Gene 1.


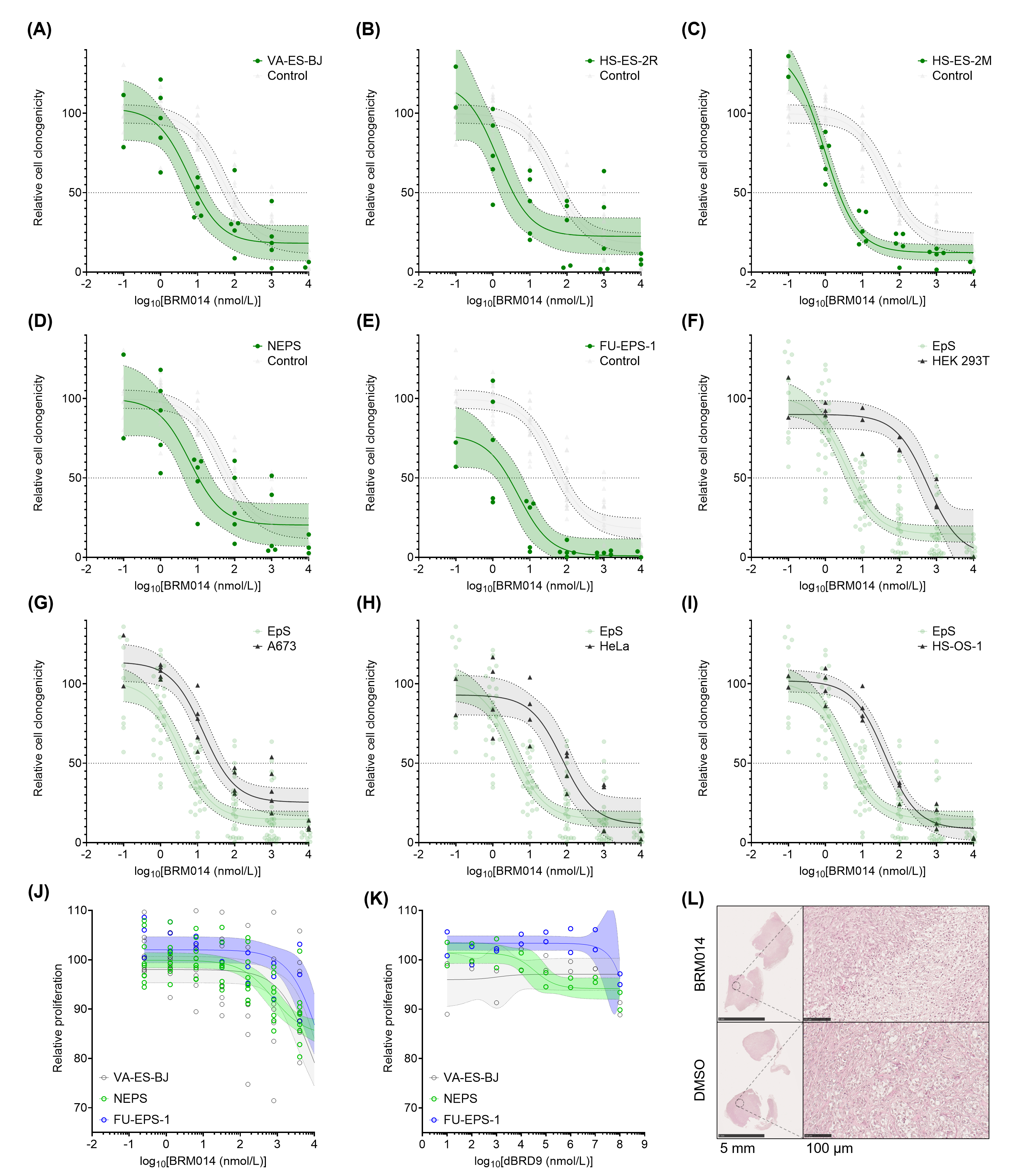
**Supplementary Figure S6.** **Drug assays reveal a significant therapeutic window for rSWI/SNF targeting in EpS.**

**(A-I)** Individual BRM014 drug response curves of SWI/SNF deficient EpS and control cell lines, calculated from clonogenicity (colony formation) assays, show a greatly increased susceptibility of EpS cell lines to (r)SWI/SNF inhibition compared to other SWI/SNF-proficient cell lines.

**(J-K)** Resazurin metabolization-based 48-hour drug response curves with 95% confidence intervals in EpS cell lines for BRM014 (J) and dBRD9 (K), demonstrating modest short-term effects of BRM014 and insignificant short-term effects of dBRD9 at clinically relevant dosages.

**(L)** Cross-sectional H&E-stained VA-ES-BJ xenograft images after treatment with BRM014 or DMSO vehicle control, showing increased levels of core necrosis in the BRM014 treatment group. Black bars on the lower left indicate 5 mm for overviews (left) and 100 µm for close-ups (right).

Abbreviations: (r)SWI/SNF, (residual) SWItch/Sucrose Non-Fermentable; EpS, Epithelioid Sarcoma; H&E, Hematoxylin and eosin; DMSO, Dimethyl sulfoxide.

**
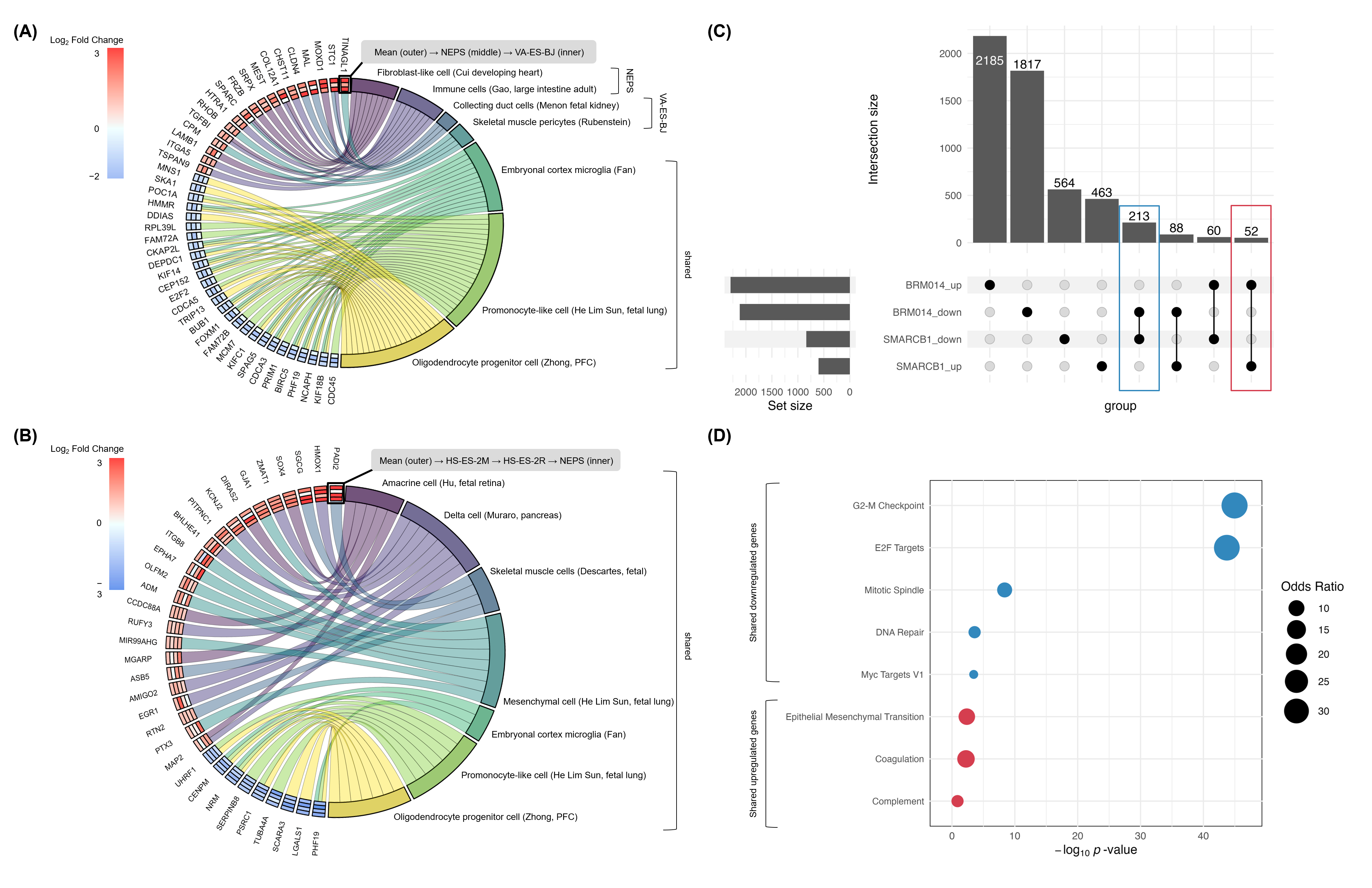
**

**Supplementary Figure S7.** **Both *SMARCB1* re-expression and SWI/SNF inhibition lead to downregulation of immature cell signatures, cell cycle progression, and proliferation while promoting differentiation/EMT processes.**

**(A)** Chord diagram showing the core genes of differentially regulated GSEA-based cell signatures for NEPS and VA-ES-BJ upon *SMARCB1* re-expression. This diagram represents log_2_ fold changes as different annuli on the left (from outermost to innermost: Mean, NEPS, VA-ES-BJ), with cell signatures shown on the right. Connections from left to right indicate gene membership in the leading-edge analysis of a signature.

**(B)** Chord diagram showing the core genes of significantly regulated cell signatures for NEPS and VA-ES-BJ upon BRM014 treatment. Log_2_ fold changes are shown as different annuli on the left (from outermost to innermost: Mean, HS-ES-2M, HS-ES-2R, NEPS), with cell signatures shown on the right. Connections from left to right indicate gene membership in the leading-edge analysis of a signature.

**(C)** Upset plot showing the intersections of differentially expressed genes upon *SMARCB1* re-expression and BRM014 treatment. Shared downregulated genes are highlighted in blue, while shared upregulated genes are highlighted in red.

**(D)** EnrichR-based, MSigDB hallmark (2020) gene set enrichment analysis of the top regulated gene sets found in shared downregulated (blue) and shared upregulated (red) genes. This analysis demonstrates shared downregulation of cell cycle progression and proliferation-associated gene sets in favor of epithelial-mesenchymal transition processes.

Abbreviations: GSEA, Gene set enrichment analysis; SMARCB1, SWI/SNF-related matrix-associated actin-dependent regulator of chromatin subfamily B member 1.

**
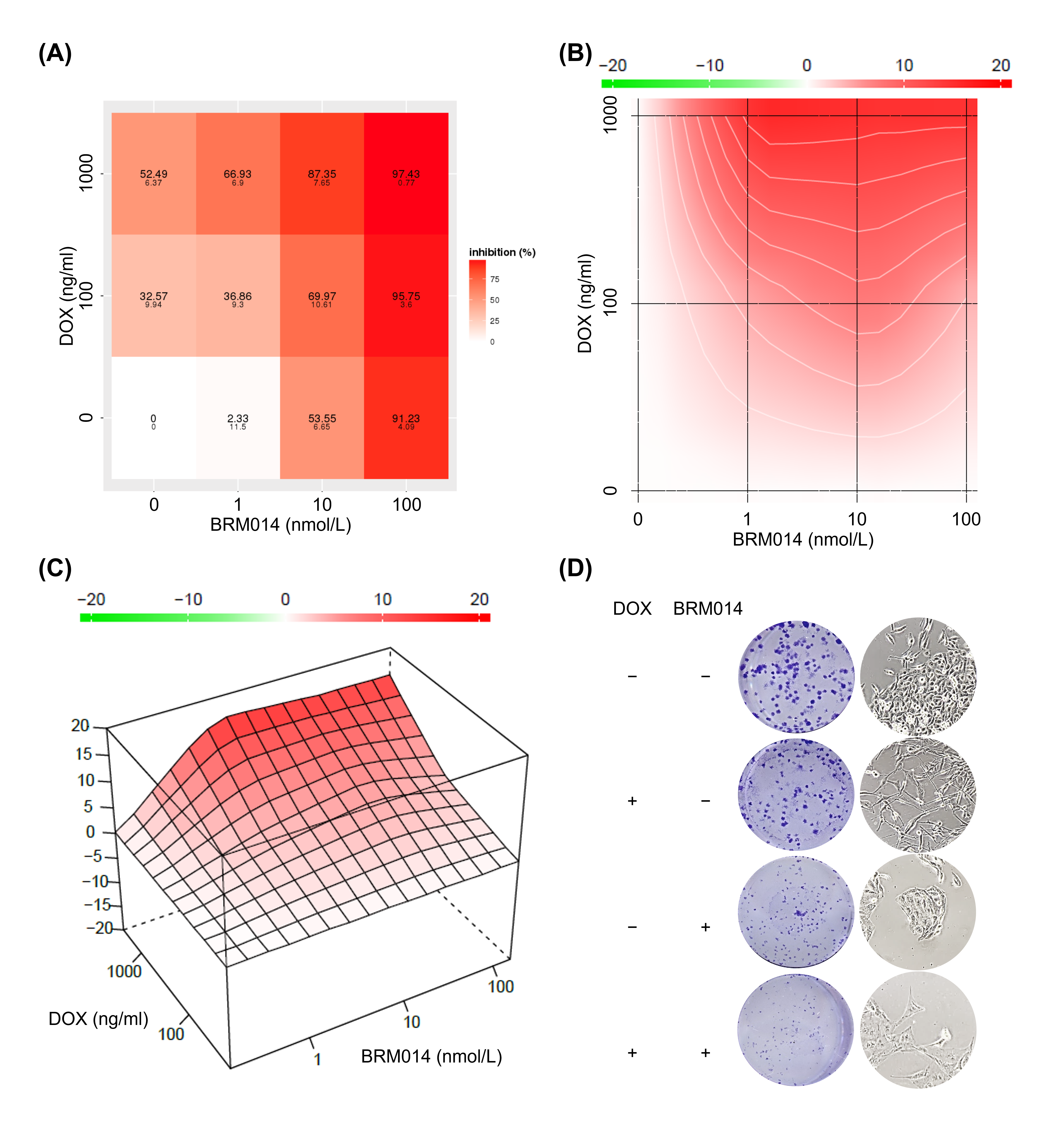
**

**Supplementary Figure S8.** ***SMARCB1* re-expression and rSWI/SNF inhibition cooperate synergistically to reduce clonogenicity.**

**(A)** Dose-response matrix of DOX × BRM014 drug synergy assays with means and standard error of the mean for every combination in VA-ES-BJ (*n* = 6 biological replicates), derived using the SynergyFinder tool, demonstrating the cooperative functionality of *SMARCB1* re-expression and rSWI/SNF inhibition.

**(B)** Bliss synergy scores for the drug response seen in (A), highlighting synergistic action at lower dose combinations and effect saturation at higher dose combinations of *SMARCB1* re-expression and rSWI/SNF inhibition, derived using the SynergyFinder tool.

**(C)** 3D Bliss synergy plot of the drug response shown in (A), emphasizing a local maximum of synergistic action at the dose combination of 100 ng/ml DOX × 1 nmol/L BRM014, derived using the SynergyFinder tool.

**(D)** Exemplary clonogenicity assay wells (100 ng/ml DOX × 100 nmol/L BRM014) alongside brightfield imaging in VA-ES-BJ, showing the co-existence of epithelioid and mesenchymal-like cells at baseline with a shift towards mesenchymal-like cell morphology upon *SMARCB1* re-expression. This shift is also observed during combination treatment, albeit to a lesser degree.

**Supplementary Table S1. Primer sequences and purpose.**

| **Name** | **Sequence** | **Purpose** |
| --- | --- | --- |
| CCND1-CTCF_Fw | 5'-GGTGGGAGGTCTTTTTGTTTC-3' | ChIP-PCR |
| CCND1-CTCF_Rv | 5'-CACGCAATCCCAGATCAAAAC-3' | ChIP-PCR |
| CCN1_E1_F | 5'-AGCTTGTTGGCGTCTTCG-3' | ChIP-PCR |
| CCN1_E1_R | 5'-TGGAGAAGGGTGACGACTAAG-3' | ChIP-PCR |
| CDKN1A_Fw | 5'-ACTGACTCATCACTACTCCCTC-3' | ChIP-PCR |
| CDKN1A_Rv | 5'-GTGTGCTATTCCCGCCAG-3' | ChIP-PCR |
| CUL1_Fw | 5'-TCCTCCCTTCTAGAAAGAGCTGAC-3' | ChIP-PCR |
| CUL1_Rv | 5'-AGGCCCGAAACCACAGAGCATAAA-3' | ChIP-PCR |
| FOSB_E4_F | 5'-TCTCCCTCTGTCTTTTCTCCT-3' | ChIP-PCR |
| FOSB_E4_R | 5'-ATCTCATGTCCCCAACGAAC-3' | ChIP-PCR |
| FOSL2_E1_F | 5'-CCACATCTCCCCTCTTTACTG-3' | ChIP-PCR |
| FOSL2_E1_R | 5'-TTTTCTCTGCTCCACACCTG-3' | ChIP-PCR |
| FUT4_E1_F | 5'-ACCAACTGAGCCAACATGTG-3' | ChIP-PCR |
| FUT4_E1_R | 5'-GAGTTCTCGAAAGCCAGGTAG-3' | ChIP-PCR |
| GATA2 _Fw | 5'-CTCAGGACCCATGGAAGTATTG-3' | ChIP-PCR |
| GATA2_Rv | 5'-CTGCAATCCTCTTAGCCTCTAG-3' | ChIP-PCR |
| IGF2_Fw | 5'-AGTGCTCGGAATGTTTGGGAACTG-3' | ChIP-PCR |
| IGF2_Rv | 5'-AGTTACCAGGAGGTGCTCAAGTGT-3' | ChIP-PCR |
| SCRT2_E1_F | 5'-TTTATATGGGACGCAGGCTG-3' | ChIP-PCR |
| SCRT2_E1_R | 5'-TTGATGGATTAGAGCGCCG-3' | ChIP-PCR |
| Myco-F1 | 5'-ACACCATGGGAGCTGGTAAT-3' | Mycoplasma PCR |
| Myco-F1t | 5'-ACACCATGGGAGCTGGTAAT-3' | Mycoplasma PCR |
| Myco-F2 | 5'-GTTCTTTGAAAACTGAAT-3' | Mycoplasma PCR |
| Myco-F2a | 5'-ATTCTTTGAAAACTGAAT-3' | Mycoplasma PCR |
| Myco-F2cc | 5'-GCTCTTTCAAAACTGAAT-3' | Mycoplasma PCR |
| Myco-R1ac | 5'-CTTCATCGACTTCCAGACCCAAGGCAT-3' | Mycoplasma PCR |
| Myco-R1cat | 5'-CCTCATCGACTTTCAGACCCAAGGCAT-3' | Mycoplasma PCR |
| Myco-R1tt | 5'-CTTCTTCGACTTTCAGACCCAAGGCAT-3' | Mycoplasma PCR |
| Myco-R2 | 5'-GCATCCACCAAAAACTCT-3' | Mycoplasma PCR |
| Myco-R2at | 5'-GCATCCACCAAATACTCT-3' | Mycoplasma PCR |
| Myco-R2ca | 5'-GCATCCACCACAAACTCT-3' | Mycoplasma PCR |
| Myco-R1 | 5'-CTTCATCGACTTTCAGACCCAAGGCAT-3' | Mycoplasma PCR |
| AgeI_SMARCB1_FW | 5'-ATTAACCGGTGCCACCATGATGATGATGGCGCT-3' | Touchdown PCR |
| SMARCB1_NotI RV | 5'-TAATGCGGCCGCTTACCAGGCCGGGG-3' | Touchdown PCR |
| M13_seq_F | 5'-CGTTGTAAAACGACGGCCAGT-3' | pENTR233 sequencing |
| M13_seq_R | 5'-CCAGGAAACAGCTATGAC-3' | pENTR233 sequencing |
| pTP_Seq_AgeI_F | 5'-ACGTATGTCGAGGTAGGCGT-3' | pTP sequencing |
| pTP_Seq_NotI_R | 5'-TTCGTCTGACGTGGCAGC-3' | pTP sequencing |
| RPLPO_F | 5'-GAAACTCTGCATTCTCGCTTC-3' | qRT-PCR |
| RPLPO_R | 5'-GGTGTAATCCGTCTCCACAG-3' | qRT-PCR |
| SMARCB1_E4_F (exon 4-5) | 5'-ACTTAGATGCCGTGCCATG-3' | qRT-PCR |
| SMARCB1_E4_R (exon 4-5) | 5'-TGCGTTCTCATGGATCACAG-3' | qRT-PCR |
| SMARCB1_E8_F (exon 8-3’UTR) | 5'-GACGCTGAGATGGAGAAGAAG-3' | qRT-PCR |
| SMARCB1_E8_R (exon 8-3’UTR) | 5'-CTTCTGAGATGCTCCGTGG-3' | qRT-PCR |
| SMARCB1_E4_TaqMan | 5'-ATGCGGTTCCTGTTGATGGTTGTG-3' | qRT-PCR TaqMan probe |
